# Supplementary material for: Chronic Viral Infection Promotes Efficient Germinal Center B Cell Responses
Source: Cell Rep. 2020 Jan 28;30(4):1013–1026.e7. doi: 10.1016/j.celrep.2019.12.023 (PMC6996002; doi:10.1016/j.celrep.2019.12.023)
Supplement: Document S1. Figures S1–S5 [file mmc1.pdf]

Cell Reports, Volume *30*

Supplemental Information

Chronic Viral Infection Promotes Efficient

Germinal Center B Cell Responses

Bénédict Fallet, Yi Hao, Marianna Florova, Karen Cornille, Alba Verge de los Aires, Giulia Girelli Zubani, Yusuf I. Ertuna, Victor Greiff, Ulrike Menzel, Karim Hammad, Doron Merkler, Sai T. Reddy, Jean-Claude Weill, Claude-Agnès Reynaud, and Daniel D. Pinschewer

Supplementary figure 1

```
WEN-1 HC
1        10        20        30        40        50        60        70        80        90        100       110       120       130
|        |         |         |         |         |         |         |         |         |         |         |         |         |
‹-----------FR1---------›‹-CDR1-›‹------FR2------›‹-CDR2-›‹----------------FR3-----------------›‹----------CDR3---------›‹---JH----›
                              S                                 K          S    M                                   AM Y
QVQLQQSGPELVKPGASVKISCKASGYAFSNSWMNWVKQRPGKGLEWIGRIYPGDGDTNYNGKFRGKATLTADKSFSTAYIQLSSLTSEDSAVYFCARNIVGEGLYYDYDGGELFDYTVDCWGQGTSVTVSS

WEN-1 LC
1        10        20        30        40        50        60        70        80        90        100       110
|        |         |         |         |         |   CDR   |         |         |         |         |         |
‹------------FR1---------›‹--CDR1--›‹------FR2------›‹2›‹--------------FR3-----------------›‹--CDR3-›‹---JK---›
                             D   I   N               A                           M            S           K
DIVLTQSPASLAVSLGQRATISCRASESVYNYGFSFMSWFQQKPGQPPKLLIYVASNQGSGVPARFSGSGSGTDFSLNIHPVEEDDTAMYFCQQTKEVPFTFGSGTNLEIK


WEN-3 HC
1        10        20        30        40        50        60        70        80        90        100       110       120
|        |         |         |         |         |         |         |         |         |         |         |         |
‹-----------FR1---------›‹-CDR1-›‹------FR2------›‹-CDR2-›‹----------------FR3-----------------›‹----CDR3----›‹---JH----›
                       A  YT    Y N     P            GS NT    K     T   E   S   M     T
QVQLKQSGAELVRPGASVKLSCKTSGFIFTDYWIHWVKQRSGQGLEWIARIYPRTGTAYYNENFKGKAVLTADKSSNTAYLQLSSLKSEDSAVYFCARWRWPREVYNMDYWGQGTSVTVSS

WEN-3 LC
1        10        20        30        40        50        60        70        80        90        100       110
|        |         |         |         |         |   CDR   |         |         |         |         |         |
‹------------FR1---------›‹--CDR1--›‹------FR2------›‹2›‹----------------FR3---------------›‹--CDR3-›‹---JK---›
                          E  D YG   M      P                                    P              N             I
DIVLTQSPASLAVSLGQRATISCRASKSVASSDNSFLHWYQQKLGQPPKLLIYRASNLESGIPARFSGSGSRTDFTLTINSVEADDVATYYCQQSYEDPFTFGSGTKLELK
```

Supplementary Figure 1 (related to Figure 1): V(D)J amino acid sequences of the LCMV-neutralizing monoclonal antibodies WEN-1 and WEN-3 in comparison to their respective unmutated ancestors

Heavy and light chain (HC, LC) sequences are represented, with changes from their V_H/J_H and $V\kappa/J\kappa$ germline counterparts indicated above the sequence. CDR regions (IMGT nomenclature) are indicated in bold. The WEN-1 HC (IGHV1-82/D2-4/JH4) displays 8 nucleotide (and 7 amino acid) changes from germline with an unusual 25 amino acid-long CDR3; the WEN-3 HC (IGHV1-76/D2-3/JH4) has 33 nucleotide exchanges and 16 amino acid mutations. The WEN-1 LC (IGKV3-2/IGKJ4) harbors 12 nucleotide and 7 amino acid changes, and the WEN-3 LC (IGKV3-5/IGKJ4) comprises 13 nucleotide and 9 amino acid changes.

Supplementary figure 2

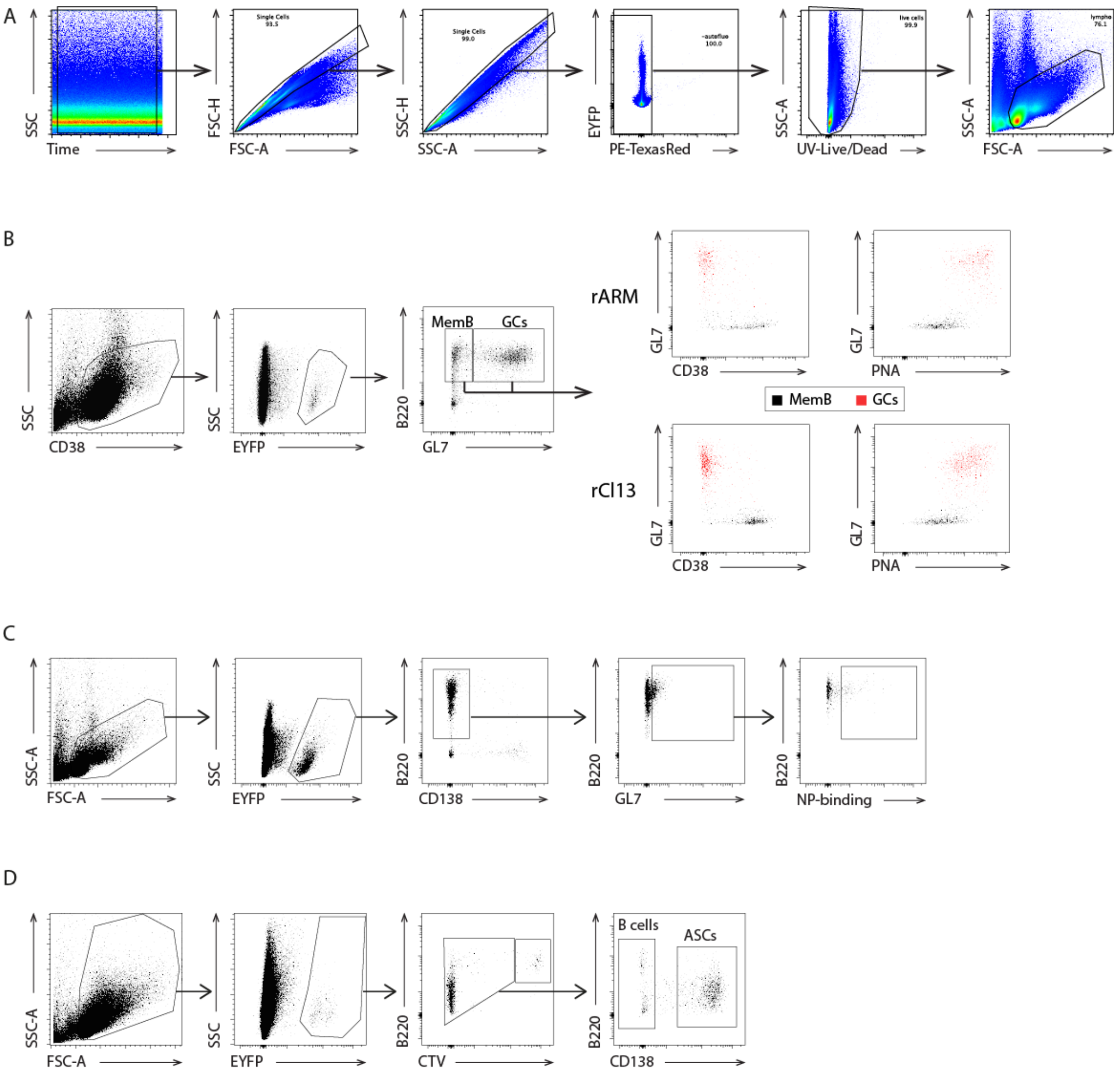

Supplementary Figure 2 (related to Figure 2): General gating strategy for viable lymphocytes, characterization of EYFP-labeled GL7$^+$B220$^+$ GC B cells and GL7$^-$B220$^+$ memory B cells, and gating strategy for the FACS analyses described in Figure 2

A. For all experiments in this manuscript lymphocytes were gated as described here: The first and last few events were gated out in a Time vs SSC gate. Doublets were then excluded based on FSC-A vs FSC-H and SSC-A vs SSC-H profiles. The PE-TexasRed channel was kept free and was used to exclude autofluorescent cells. Dead cells were stained as described in Methods and were gated out as shown. Lymphocytes were then gated based on forward- and side-scatter. All gating strategies in subsequent Supplementary figures and panels are based upon the analogous pre-gating strategy.

B: We infected AID^{rep} mice either rARM or rCl13 on day 0 and treated them with tamoxifen on day 0 and day 5. On day 35 we characterized PNA and CD38 profiles of EYFP-expressing B220$^+$GL7$^+$ GC B cells and of EYFP$^+$ B220$^+$GL7$^-$ MemB cells. Representative FACS plots from one out of four mice are shown except for GL7/CD38 and GL7/PNA plots, which display the combined events from two representative out of four mice.

C. We infected AID^{rep} mice with rCl13 or VSV on day 0 or left them untreated. Tamoxifen was administered on day 0, 5 and 10 as described for Fig. 2A. The gating strategy as shown was applied to quantify NP-binding cells.
D. We transferred Vacc-experienced or rCl13-experienced B cells into syngeneic C57BL/6 recipients, which had been infected with rCl13 six days before or with Vacc three days before, respectively, as described in Fig. 2D. The gating strategy as shown was used to enumerate proliferated CTV^{lo} $EYFP^{+}$ B cells and ASCs.
Representative FACS plots are shown.

Supplementary figure 3

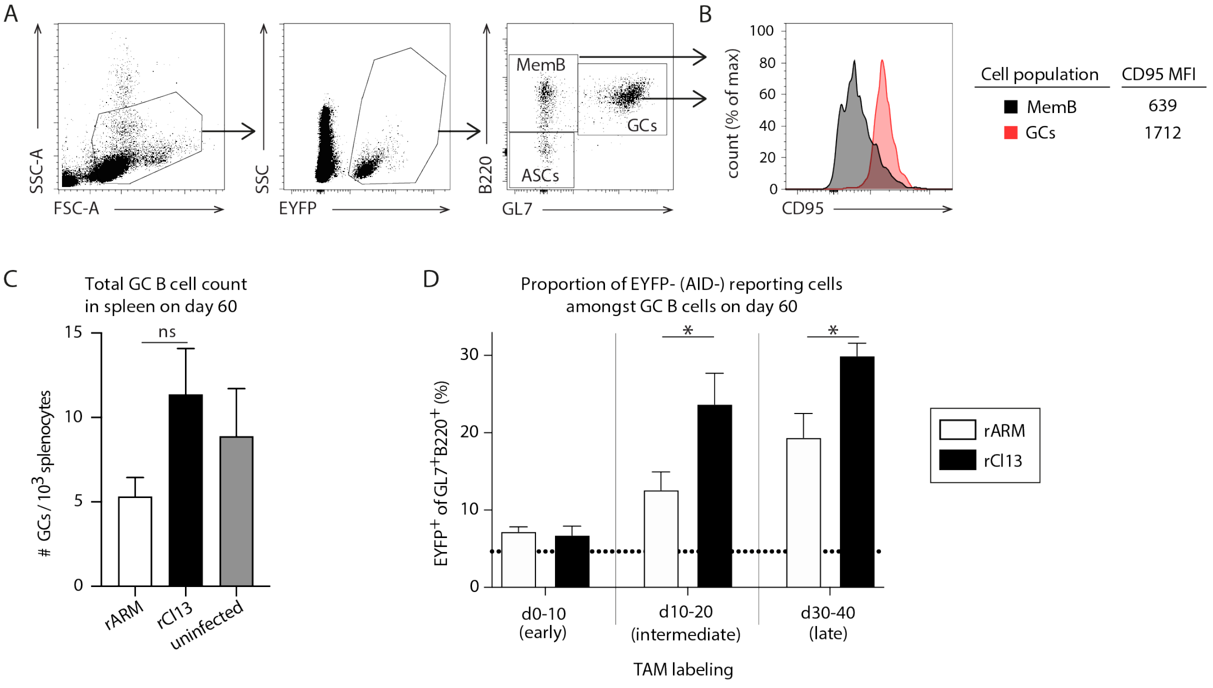

Supplementary Figure 3 (related to Figure 3): Gating strategy for the FACS analyses shown in Figure 3, total GC B cell counts and representation of EYFP-reporting GC B cells in spleens of rARM- and rCl13-infected mice on day 60.

A,B: We infected AID^{rep} mice with rARM or rCl13, followed by tamoxifen labeling on day 0 and 5 (early), 10 and 15 (intermediate) or 30 and 35 (late) after infection as described in Fig 3A. The gating strategy as displayed in (A) was used to quantify EYFP^{+} GC B cells, MemB cells and ASCs on day 60 after infection. The CD95 expression profile of EYFP-expressing B220^{+}GL7^{+} cells in comparison to EYFP^{+} B220^{+}GL7^{-} MemB cells was analyzed in (B). Representative FACS plots are shown.

C: We infected mice with rCl13 or rARM and left controls uninfected. Total GL7^{+}B220^{+} GC B cells were enumerated in spleen 60 days later. One-way ANOVA with Bonferroni's post-test for multiple comparisons. ns: $p > 0.05$.

D: EYFP^{+} (AID-reporting) GL7^{+}B220^{+} GC B cells from Fig. 3C are displayed as a proportion of the total GL7^{+}B220^{+} GC B cell compartment. Bars in (C,D) represent means +/- SEM. The dotted line indicates background EYFP reporting cells in uninfected control mice. n = 4 to 5, N = 2. Two-way ANOVA with Bonferroni's post-test for multiple comparisons. *: $p < 0.05$. Data in (C,D) are from the same experiment as reported in the main Fig. 3C.

Supplementary figure 4

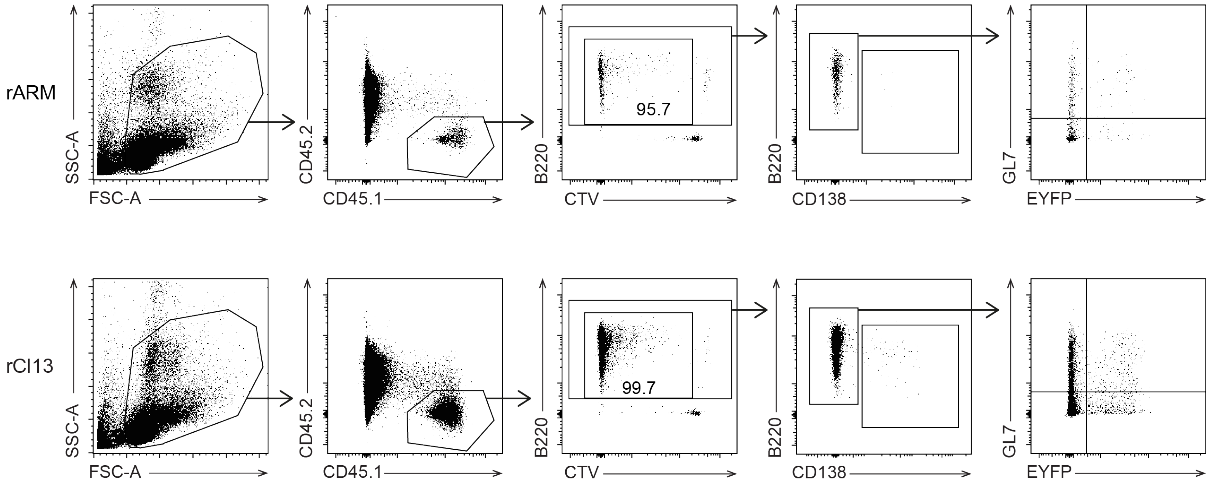

Supplementary Figure 4 (related to Figure 4): Gating strategy for the FACS analyses shown in Figure 4

We infected wt recipient mice with rARM* or rCl13*. On day 20 after infection these animals were given KL25HL-AID^{rep} B cells by adoptive transfer and were administered TAM on day 20 and 23 as shown in Fig 4A. The gating strategy as displayed was used to determine EYFP and GL7 expression by the transferred KL25HL-AID^{rep} B cells. Numbers in FACS plots indicate the percentage of CTV^{lo} cells amongst transferred B220^{+} cells. Representative FACS plots are shown.

Supplementary figure 5

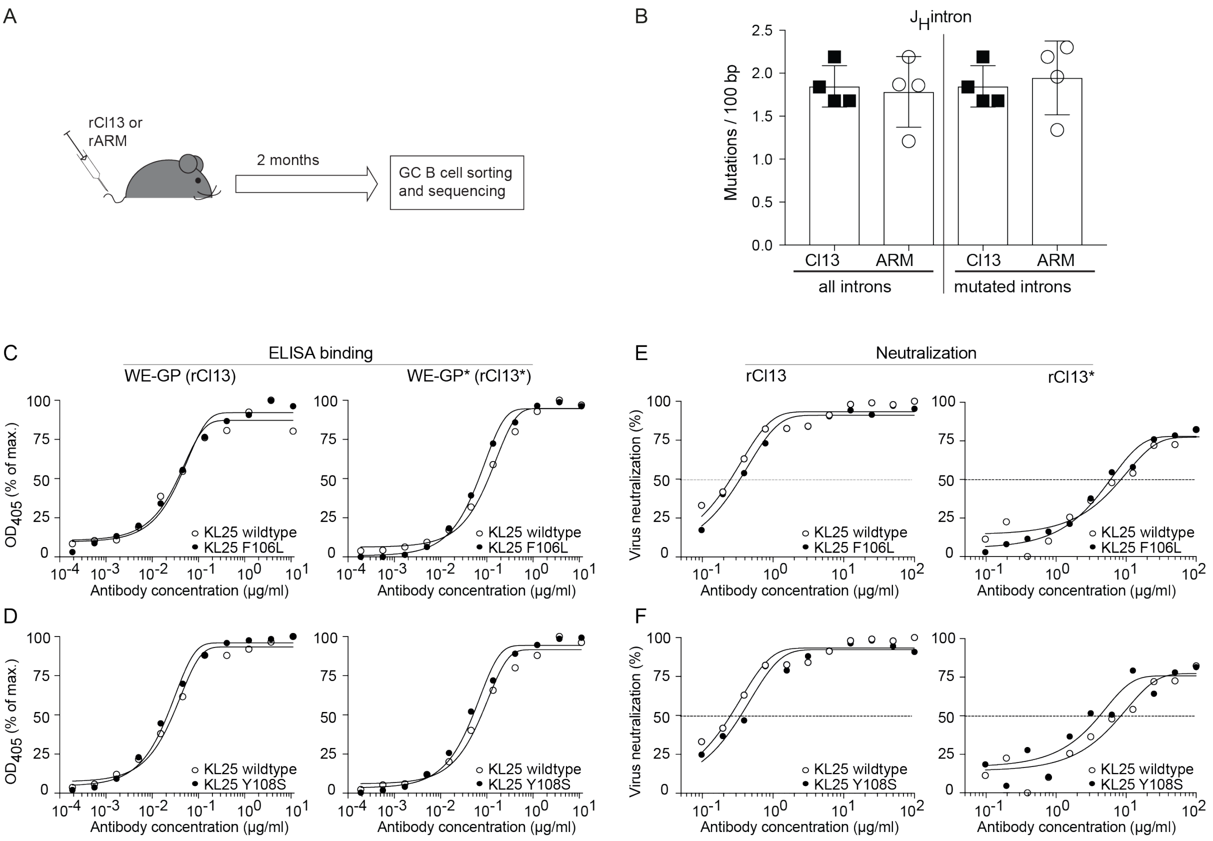

Supplementary Figure 5 (related to Figure 5): Comparable mutational load in V_H introns of GC B cells from rARM- and rCl13-infected mice and characterization of KL25 variants selected in rCl13* infection

A,B: We infected wt mice with rCl13 or rARM. Two month later we sorted $CD3^{-}$ IgD^{-} IgM^{-} $GL7^{+}$ $B220^{+}$ B cells from the spleen for IgH intron sequencing as described in Methods. Schematic of the experimental design (A). Mutations frequencies in the J_H intron are shown as nucleotide changes per 100 bp as calculated for all sequences (left) or for mutated sequences only (right), (B). Bars represent mean +/- SD. Symbols represent individual mice. n = 4, N = 1.

C,D: Binding of KL25 wildtype, KL25-Y108S and KL25-F106L to WE-GP and WE-GP*.

E,F: Neutralization of rCl13 and rCl13* by KL25 wildtype, KL25-Y108S and KL25-F106L. Symbols in (C-F) show the mean of 2 technical replicates. N = 2 (D,E).
