# Supplementary material for: Chronic Viral Infection Promotes Efficient Germinal Center B Cell Responses
Source: Cell Rep. 2020 Jan 28;30(4):1013–1026.e7. doi: 10.1016/j.celrep.2019.12.023 (PMC6996002; doi:10.1016/j.celrep.2019.12.023)
Supplement: Document S2. Article plus Supplemental Information [file mmc2.pdf]

Cell Reports

Article

Chronic Viral Infection Promotes Efficient Germinal Center B Cell Responses

Graphical Abstract

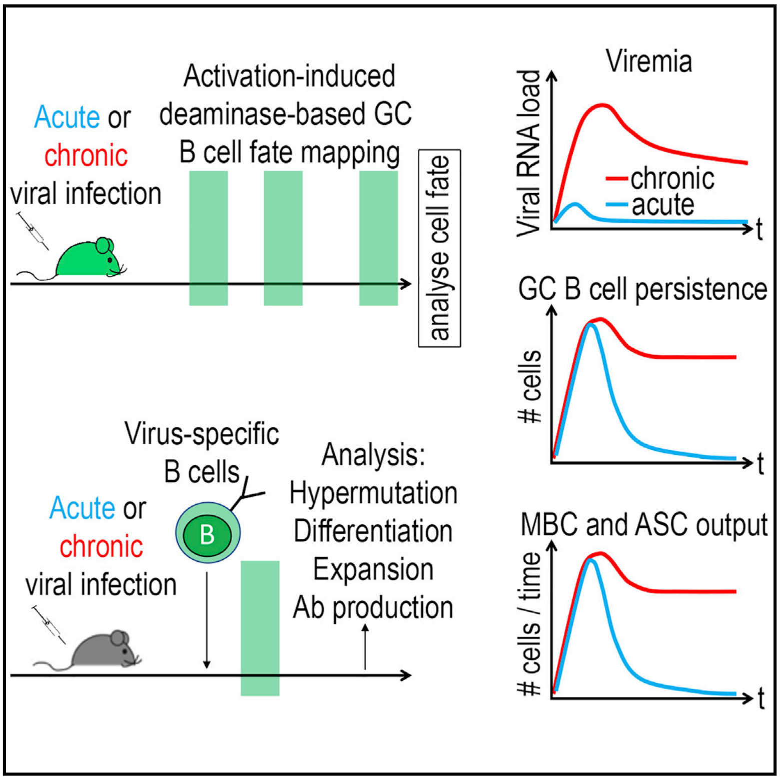

Highlights

- Chronic viral infection elicits potent and sustained germinal center (GC) responses
- Chronic infection triggers prolonged plasma cell and memory B cell output from GCs
- GC B cells hypermutate efficiently and are potently selected in chronic infection

Authors

Bénédict Fallet, Yi Hao, Marianna Florova, ..., Jean-Claude Weill, Claude-Agnès Reynaud, Daniel D. Pinschewer

Correspondence

daniel.pinschewer@unibas.ch

In Brief

Fallet et al. used AID fate-mapping to compare germinal center responses to chronic and acute viral infection. Germinal center B cells in chronic infection hypermutate efficiently, adapt to viral variants, and yield more antibody-secreting cells and memory B cells for longer time periods than found in acute infection, enabling neutralizing antibody formation.

Fallet et al., 2020, Cell Reports *30*, 1013–1026

January 28, 2020 © 2019 The Authors.

https://doi.org/10.1016/j.celrep.2019.12.023

CellPress

Cell Reports
Article

OPEN ACCESS
CellPress

Chronic Viral Infection Promotes Efficient Germinal Center B Cell Responses

Bénédict Fallet,[1,7] Yi Hao,[2,3,7] Marianna Florova,[1,7] Karen Cornille,[1] Alba Verge de los Aires,[2] Giulia Girelli Zubani,[2] Yusuf I. Ertuna,[1] Victor Greiff,[4,5] Ulrike Menzel,[4] Karim Hammad,[6] Doron Merkler,[6] Sai T. Reddy,[4] Jean-Claude Weill,[2,8] Claude-Agnès Reynaud,[2,8] and Daniel D. Pinschewer[1,8,9,*]
[1]Department of Biomedicine, Division of Experimental Virology, University of Basel, Haus Petersplatz, 4009 Basel, Switzerland
[2]Development of the Immune System, Institut Necker-Enfants Malades, Institut National de la Santé et de la Recherche Médicale, U1151-Centre National de la Recherche Scientifique, UMR 8253, Faculté de Médecine Paris Descartes, Université Paris Descartes, Sorbonne Paris Cité, Paris, France
[3]Department of Pathogen Biology, School of Basic Medicine, Tongji Medical College, Huazhong University of Science and Technology, Wuhan, China
[4]Department of Biosystems Science and Engineering, ETH Zürich, Basel, Switzerland
[5]Department of Immunology, University of Oslo, Oslo, Norway
[6]Department of Pathology and Immunology, Division of Clinical Pathology, University & University Hospital of Geneva, Geneva, Switzerland
[7]These authors contributed equally
[8]These authors contributed equally
[9]Lead Contact
*Correspondence: daniel.pinschewer@unibas.ch
https://doi.org/10.1016/j.celrep.2019.12.023

SUMMARY

Persistent viral infections subvert key elements of adaptive immunity. To compare germinal center (GC) B cell responses in chronic and acute lymphocytic choriomeningitis virus infection, we exploit activation-induced deaminase (AID) fate-reporter mice and perform adoptive B cell transfer experiments. Chronic infection yields GC B cell responses of higher cellularity than acute infections do, higher memory B cell and antibody secreting cell output for longer periods of time, a better representation of the late B cell repertoire in serum immunoglobulin, and higher titers of protective neutralizing antibodies. GC B cells of chronically infected mice are similarly hypermutated as those emerging from acute infection. They efficiently adapt to viral escape variants and even in hypermutation-impaired AID mutant mice, chronic infection selects for GC B cells with hypermutated B cell receptors (BCRs) and neutralizing antibody formation. These findings demonstrate that, unlike for $CD8^+$ T cells, chronic viral infection drives a functional, productive, and protective GC B cell response.

INTRODUCTION

Persistent viral diseases, such as HIV, hepatitis B virus (HBV), and hepatitis C virus (HCV) infection, represent major global health challenges and affect several hundred million people worldwide (WHO, 2018a, 2018b, 2018c). A commonly held concept suggests that viral persistence requires subversion of the host's adaptive immune defense. $CD8^+$ T cell exhaustion, caused by chronic antigenic stimulation, represents a paradigmatic example (Gallimore et al., 1998; Moskophidis et al., 1993; Zajac et al., 1998). In contrast to T cells, the effects of chronic antigenic stimulation on B cell responses to persisting viruses remain less well defined. In chronic HBV and HIV infection, the B cell compartment is subject to phenotypic alterations, with an accumulation of atypical $CD21^{neg}CD27^{neg}$ memory B cells and plasmablasts in peripheral blood (Burton et al., 2018; Moir and Fauci, 2014). In these patients, circulating antiviral B cells express a range of inhibitory receptors, such as FcRL4 and PD-1 (Burton et al., 2018; Moir et al., 2008; Salimzadeh et al., 2018). Moreover, HBV-specific B cells of chronically infected patients fail to differentiate into antibody-secreting cells (ASCs) upon *in vitro* re-stimulation and produce inadequate amounts of immunoglobulin, both of which can be partially restored by PD-1 blockade (Burton et al., 2018; Salimzadeh et al., 2018). Impaired antibody responses to vaccination with third-party antigens (Malaspina et al., 2005) and a shortened life span of memory B cells (Wheatley et al., 2016) can be interpreted to reflect generalized suppression of the humoral immune system in HIV-infected individuals. Similarly, chronic lymphocytic choriomeningitis virus (LCMV) infection in mice is associated with suppressed antibody responses to third-party antigens (Bergthaler et al., 2010; Leist et al., 1988). Counterintuitively, however, significant LCMV neutralizing antibody (nAb) responses are typically elicited under conditions of chronic infection but only rarely when acute LCMV infection is efficiently cleared (Eschli et al., 2007). Analogously, broadly neutralizing antibody (bnAb) responses to HIV itself are most commonly found in patients with long-term uncontrolled viremia (Rusert et al., 2016). These findings raised the possibility that, unlike for CD8 T cell responses, high levels of persisting viral antigen may result in an efficient antiviral germinal center (GC) B cell response. In line with this hypothesis, the spontaneous resolution of HBV infection is associated with the formation of protective anti-HBs antibodies (Guidotti et al., 2015), and evidence is accumulating that

This is an open access article under the CC BY license (http://creativecommons.org/licenses/by/4.0/).

spontaneous HCV clearance relies on the timely formation of bnAbs (Kinchen et al., 2018; Osburn et al., 2014; Pestka et al., 2007; Raghuraman et al., 2012). Of note, in this context, the envelope proteins of HIV, HCV, and LCMV represent challenging targets for antibody neutralization because of structural immune evasion features, such as prominent glycan shields (Helle et al., 2010; Sommerstein et al., 2015; Wei et al., 2003). Accordingly, these viral envelope proteins commonly fail to induce potent nAb responses when presented to the immune system in the context of vaccination (Law et al., 2013; Pinschewer et al., 2004; Rose et al., 2000; Sommerstein et al., 2015), but they do so in the context of chronic infection (Bergthaler et al., 2009; Eschli et al., 2007; Kinchen et al., 2018; Osburn et al., 2014; Pestka et al., 2007; Raghuraman et al., 2012; Richman et al., 2003; Rusert et al., 2016). Taken together, these observations raised the possibility that the humoral immune system meets the challenge of glycan-shielded antigens preferentially under conditions of chronic viremic infection. Such a response pattern—weak in vaccination and acute infection but potent in chronic infection—would seem counter-intuitive in light of the opposite findings for CD8 T cells. Only limited information is, however, available on the functional efficiency of antiviral GC B cell responses in chronic viral infection.

At the onset of LCMV infection, antiviral B cells are largely deleted because of interferon-driven inflammation, a process also referred to as ‘‘decimation’’ (Fallet et al., 2016; Moseman et al., 2016; Sammicheli et al., 2016). In light of the finding that naive B cells can readily be recruited into an ongoing antiviral response (Doria-Rose et al., 2014; Schweier et al., 2019), we and others have proposed that antiviral B cell responses in the chronic phase of infection rely on a repertoire replenishment by new bone marrow emigrants (Doria-Rose et al., 2014; Fallet et al., 2016; Zellweger et al., 2006). Pioneering studies on chronic bacterial and parasitic infections have revealed striking deviations from the ‘‘canonical’’ B cell response as it has been defined in protein-adjuvant immunizations. A dominance of very-low-affinity B cell clones at the onset of the response and their subsequent extrafollicular affinity maturation was observed in chronic murine salmonellosis (Di Niro et al., 2015). In similar violation of commonly held concepts, hypermutated immunoglobulin (Ig) M^+ memory B cells were found to dominate the recall response to *Plasmodium* parasites (Krishnamurty et al., 2016), altogether emphasizing the need to better understand how B cells respond to chronic microbial exposure.

Here, we investigated how viral persistence affects the functionality of the GC B cell response. We report that the neutralizing capacity of the murine LCMV-envelope-specific antibodies, as generated during chronic infection, requires their mutational maturation, analogous to human HIV and HCV neutralizing antibodies (Bailey et al., 2017; Georgiev et al., 2014; Jardine et al., 2016; Simonich et al., 2016; Wiehe et al., 2018; Xiao et al., 2009). Importantly, we found that chronic viremic infection drives a long-lived GC reaction with potent selection of hypermutated clones. The resulting output of memory B cells and plasma cells exceeded the cellular yields upon acute infection. In conclusion, our observations characterize the GC B cell responses underlying potent nAb formation in persistent viral infection. These insights should help to better mimic chronic infection when designing B cell-based vaccination approaches against persistent viral diseases.

RESULTS

LCMV nAbs Arise Preferentially in Chronic Infection and Require Somatic Hypermutation

To compare B cell responses in acute and chronic infection, we made use of two genetically engineered variants of LCMV, both carrying the identical LCMV-WE strain envelope glycoprotein (WE-GP) as the sole target for nAbs. The two viruses are based on either the Armstrong (rARM) or Clone 13 backbone (rCl13) and differ in a single amino acid position of the polymerase gene, which is essential for rCl13 persistence (Bergthaler et al., 2010). High-dose intravenous (i.v.) infection with rCl13 results in protracted high-level viremia, whereas rARM, administered intraperitoneally at lower doses, represents a prototypic model of acute infection without detectable viremia (Figure 1A; Bergthaler et al., 2007; Sommerstein et al., 2015; Zajac et al., 1998). These two extremes of a spectrum, both in terms of antigen load and persistence, yet with identical antigenicity, allowed us to study the impact of viral chronicity on antiviral B cell responses in general and on the size and dynamics of the GC response in particular. When measuring antibodies against the highly glycosylated outer globular domain of WE-GP (GP-1) by ELISA, those developed continuously for a period of 60 days and reached significantly higher titers in rCl13- than in rARM-infected mice (Figure 1B). In addition, most rCl13-infected mice mounted nAbs by day 62, whereas rARM infection elicited detectable nAb responses in only a few animals and at lower titers (Figure 1C). Altogether, these findings confirmed earlier observations that protracted LCMV infection was a more potent driver of GP-1 binding and neutralizing Ab responses than acute infection (Eschli et al., 2007). For some acute viral infections, such as influenza virus or vesicular stomatitis virus, it has been shown that germline-encoded antibodies can potently neutralize and, accordingly, those responses can be mounted within a few days (Harada et al., 2003; Kalinke et al., 1996). Conversely, nAb responses to HIV or HCV typically take weeks to months before they arise (Chen et al., 1999; Richman et al., 2003). Isolated monoclonal nAbs are often substantially hypermutated, and the ability of unmutated ancestor antibodies to bind and/or neutralize the respective virus can be limited or undetectable (Xiao et al., 2009). In light of the finding that LCMV nAb responses arose only between days 40 and 60 after infection, we hypothesized that neutralizing activity required antibody maturation by hypermutation. To test that, we studied two LCMV-neutralizing antibodies (WEN-1 and WEN-3), which we reverted to their respective, putative, unmutated ancestor (UA) sequence (WEN-1^{UA} and WEN-3^{UA}). WEN-1 and WEN-3 display V(D)J sequence hallmarks previously reported for HIV bnAbs (Kwong and Mascola, 2012); the WEN-1 heavy chain features an extraordinary 25-amino-acid-long CDR3 but diverges from its UA by only 7 amino acid changes, whereas the heavy chain variable region (V_H) of WEN-3 stands out for its high mutational load (33 nt mutations, 16 aa divergence from its UA; Figure S1). Both WEN-1^{UA} and WEN-3^{UA} bound detectably to WE-GP but only did so at ~10- to 100-fold higher concentrations than their

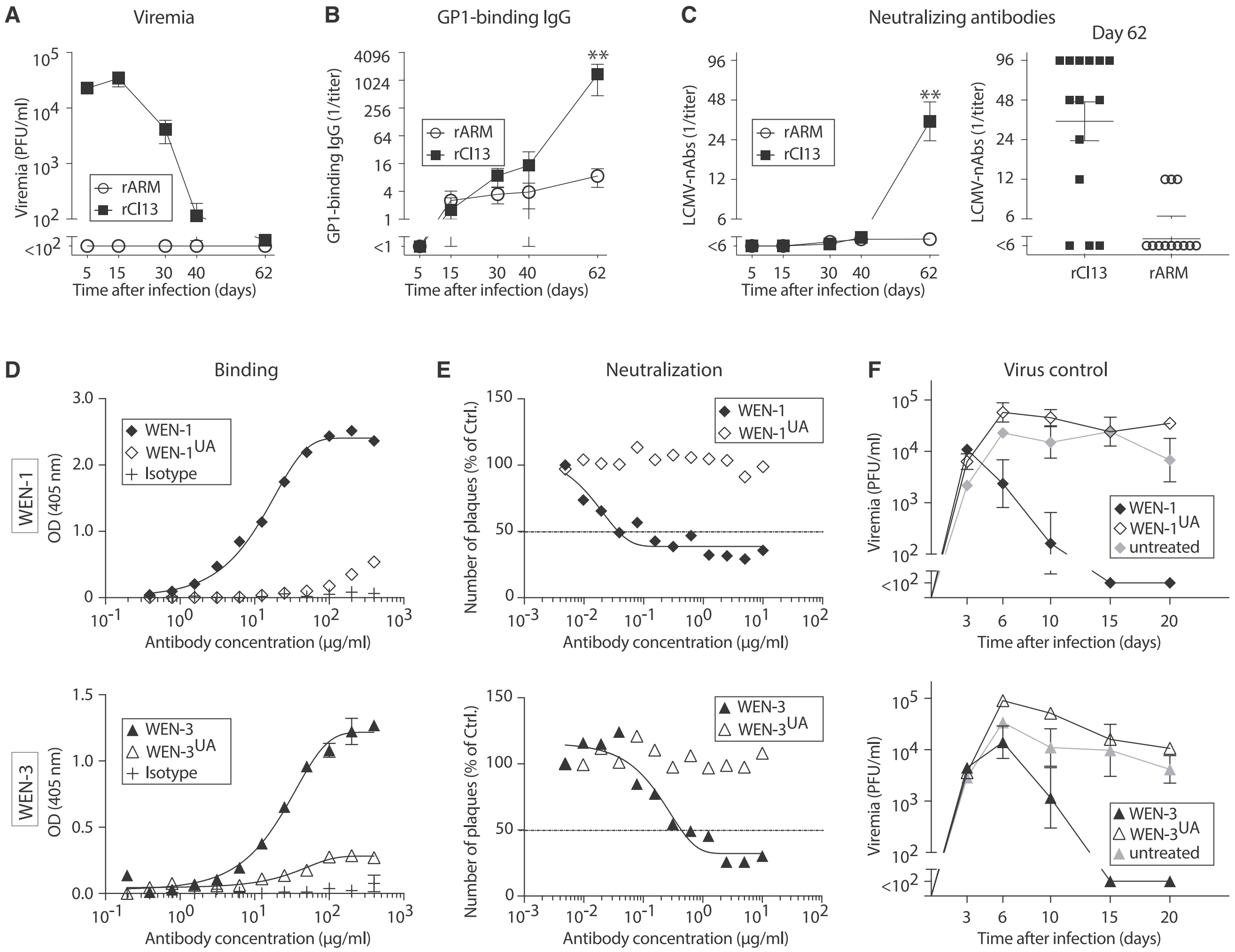

Figure 1. LCMV nAbs Arise Preferentially in Chronic Infection and Require Somatic Hypermutation

(A–C) We infected mice with rCl13 or rARM and measured viremia (A), GP1-binding IgG titers (B), and virus-neutralizing antibodies (C) from blood on the indicated days. Symbols and bars represent means ± SEM in (A), (B), and (C, left panel). The right panel in (C) shows nAb titers of individual animals on day 62. Number of biological replicates (n) = 3–15 (A), n = 5 (B), n = 12–15 (C). Number of independent experiments (N) = 3. Two-way ANOVA with Bonferroni's post-test for multiple comparisons. **p < 0.01.

(D and E) Binding of the rCl13-neutralizing antibodies WEN-1 and WEN-3, of their respective unmutated ancestors WEN-1^{UA} and WEN-3^{UA} and of an irrelevant isotype control antibody to WE-GP (D). Ability of the indicated antibodies to neutralize rCl13 (E). Symbols show the means of two technical replicates. N = 2.

(F) We infected mice with rCl13 on day 0, followed by passive immunization with the indicated antibodies on day 3. Viremia was monitored. Symbols represent the means ± SEM of three to four mice (WEN-1, one representative experiment) and of four to six mice (WEN-3, two combined experiments), respectively. N = 2. See also Figure S1.

respective hypermutated wild-type (WT) antibody counterparts (Figure 1D). In line with impaired binding, neither of the UA antibodies retained detectable virus-neutralizing capacity (Figure 1E). LCMV envelope-binding, non-neutralizing antibodies may, however, still exert protective antiviral effects (Hangartner et al., 2006). To test whether the UA antibodies may afford antiviral protection, we passively immunized rCl13-infected mice with either hypermutated or UA antibodies on day 3 after virus inoculation (Figure 1F). When either one of the hypermutated antibodies was administered, viremia was effectively suppressed, resulting in clearance of rCl13 by day 15. In contrast, neither of the UA antibodies reduced viral loads to a detectable extent, and all mice remained viremic throughout the observation period of 20 days. Taken together, these data indicated that hypermutation of LCMV glycoprotein-specific antibodies was required for potent binding, virus neutralization, and antiviral protection.

AID Reporter System Identifies LCMV-Specific B Cells in a Polyclonal Response

To investigate the underlying cellular correlate of LCMV nAb formation, we embarked on a comprehensive long-term study of the LCMV-specific GC B cell response in rCl13- and rARM-infected mice. First, we set out to test whether our AID reporter mouse model (AID^{rep} [Dogan et al., 2009]) was suitable for that

purpose. AID^{rep} mice are hemizygous for an engineered *aicda* locus (naturally encoding for activation-induced deaminase, AID), which expresses a tamoxifen (TAM)-inducible Cre recombinase (Cre-ERT2). Additionally, AID^{rep} mice carry a Cre-inducible EYFP reporter gene in the ROSA26 locus, such that TAM administration induces EYFP expression selectively in approximately 10%–20% of (AID expressing) GC B cells (Dogan et al., 2009). Thereby, timed TAM administration allows for the fate mapping of the GC B cell compartment of a given time window including these cells' progeny. Within the population of EYFP-labeled cells, we used GL7 and B220 staining to discriminate memory B cells (GL7^{-}B220^{+}), GC B cells (GL7^{+}B220^{+}), and plasma cells (GL7^{-}B220^{-}). In keeping with our earlier findings (Le Gallou et al., 2018; Thai et al., 2018), EYFP-reporting GL7^{+}B220^{+} B cells in rARM- and rCl13-infected mice were CD38$^{–}$ and bound biotinylated peanut agglutinin (PNA), identifying them as GC B cells, whereas GL7^{-}B220^{+} memory B cells exhibited an inverse staining pattern (Figures S2A and S2B). To test whether EYFP reporting identified virus-specific B cells, we infected AID^{rep} mice with rCl13, whereas control groups were given the antigenically unrelated vesicular stomatitis virus (VSV) or were left uninfected. TAM was administered to all mice on days 0, 5, and 10, with a resulting labeling window extending approximately from day 0 to day 15 after infection (Figure 2A). When analyzed on day 50, ~20% of EYFP-reporting B cells of rCl13-infected mice bound the LCMV nucleoprotein (NP) in flow cytometry (Figures 2B, 2C, and S2), which was several-fold over background of VSV-infected or uninfected controls. Given that NP represents approximately 17% of the viral proteome, these numbers were compatible with a faithful labeling of LCMV-reactive B cells in AID^{rep} mice. To further assess whether EYFP-reporting B cells in AID^{rep} mice were indeed mostly LCMV-reactive, we performed adoptive B cell transfer experiments (Figure 2D). We infected AID^{rep} mice with either rCl13 or with Vaccinia virus (Vacc) on day 0, followed by TAM treatment as above. On day 30, we isolated splenic B cells from these donor mice, containing a population of supposedly rCl13- or Vacc-specific, EYFP-labeled B cells, respectively. These (CD45.2^{+}) B cells were adoptively transferred into syngeneic (CD45.1^{+}) recipients, which had been infected with either rCl13 or Vacc beforehand. The recipients were infected ahead of the transfer, rather than afterward, to avoid the "decimation" of adoptively transferred B cells by interferon-driven inflammation (Fallet et al., 2016; Moseman et al., 2016; Sammicheli et al., 2016). When analyzed 6 days after transfer, sizeable populations of EYFP^{+} CD138$^{–}$ B cells and EYFP^{+} CD138^{+} antibody-secreting cells (ASCs) were detected in rCl13-infected recipients, but not in Vacc-infected recipients, of B cells from rCl13-infected donors and vice versa (Figures 2E and 2F). This result confirmed that AID^{rep} mice identified mostly virus-specific B cells rather than non-specifically activated polyreactive B cells, validating the AID^{rep} mouse model for our ambitions to study LCMV-specific GC B cell responses.

Chronic Infection Triggers a Sustained GC Response with Prolonged Plasma Cell and Memory B Cell Output

To compare the generation and maintenance of B cell responses in chronic and acute viral infection, we inoculated groups of AID^{rep} mice with either rCl13 or rARM and treated them with TAM in a manner corresponding to labeling windows from day 0–10 (early), day 10–20 (intermediate), or day 30–40 (late), respectively (Figure 3A). On day 60 after infection, we enumerated EYFP^{+} GL7^{+} B220^{+} GC B cells, which consistently co-expressed CD95, EYFP^{+} GL7$^{–}$ B220^{+} memory B cells (MemB cells) and EYFP^{+} GL7$^{–}$ B220$^{–}$ ASCs (Figures 3B, S3A, and S3B). Thereby, we assessed LCMV-specific B cells and plasma cells fulfilling two criteria: first, these cells or their precursors had expressed AID in the early, intermediate, and late labeling windows, respectively; and second, they were still present at the day-60 time point of analysis as GC B cells, MemB cells, or ASCs. This experimental approach did not, however, inform about the clonal relationship among cells labeled in different time windows. We found that EYFP^{+} GC B cells were approximately 3-fold more numerous in rCl13- than in rARM-infected mice, irrespective of the TAM-labeling window (Figure 3C). Conversely, the total number of GC B cells in rARM- and rCl13-infected animals was not significantly different (Figure S3C). This indicated that antiviral GC responses were maintained at lower levels in acute, as compared with chronic, infection, with a proportional reduction in cells that had expressed AID during the early, intermediate, and late labeling windows ("early labeled," "intermediate-labeled," and "late-labeled" cells). For the intermediate and late labeling windows, reduced virus-specific GC B cell responses in rARM-infected, as compared with rCl13-infected, mice were also reflected in a lower proportional representation of AID-reporting cells among total GC B cells (Figure S3D). In contrast, early labeled MemB cell (Figure 3D) and early labeled ASC (Figure 3E) pools were of comparable size in rCl13- and rARM-infected animals, suggesting that in the early phase after virus inoculation, the MemB cell and ASCs output of acute and chronic infection was comparable. With transition from the early to intermediate and late labeling windows, however, the MemB cell and ASC output of rARM-infected mice declined progressively, which contrasted with sustained levels in rCl13 infection. These observations found independent support in immunohistochemical analyses from spleens of early labeled mice, showing that EYFP^{+} cells inside splenic GCs were more numerous in rCl13 than in rARM infection (Figure 3F). Taken together, these observations indicated that GC responses in chronic infection were sustained at higher levels over time and, in the long term, drove higher MemB cell and ASC output than acute infection.

Chronic Infection Is a Potent Long-term Driver of B Cell Responses

We aimed to corroborate the hypothesis that at intermediate to late time points after virus inoculation, chronic infection was a more potent driver of B cell responses than was acute infection. For that, we performed adoptive transfer experiments with Cell Trace Violet (CTV)-labeled B cells of AID^{rep} mice, which express the monoclonal LCMV envelope-specific B cell receptor KL25 from a heavy chain knockin and a light chain transgene (KL25HL-AID^{rep} mice, CD45.1; Figure 4A). Twenty days before transfer, the syngeneic (CD45.2) recipients were infected with genetically engineered rCl13 or rARM variants (rCl13*, rARM*) carrying the N121K point mutation in their WE-GP envelope protein (WE-GP*). This envelope variant is only poorly neutralized by

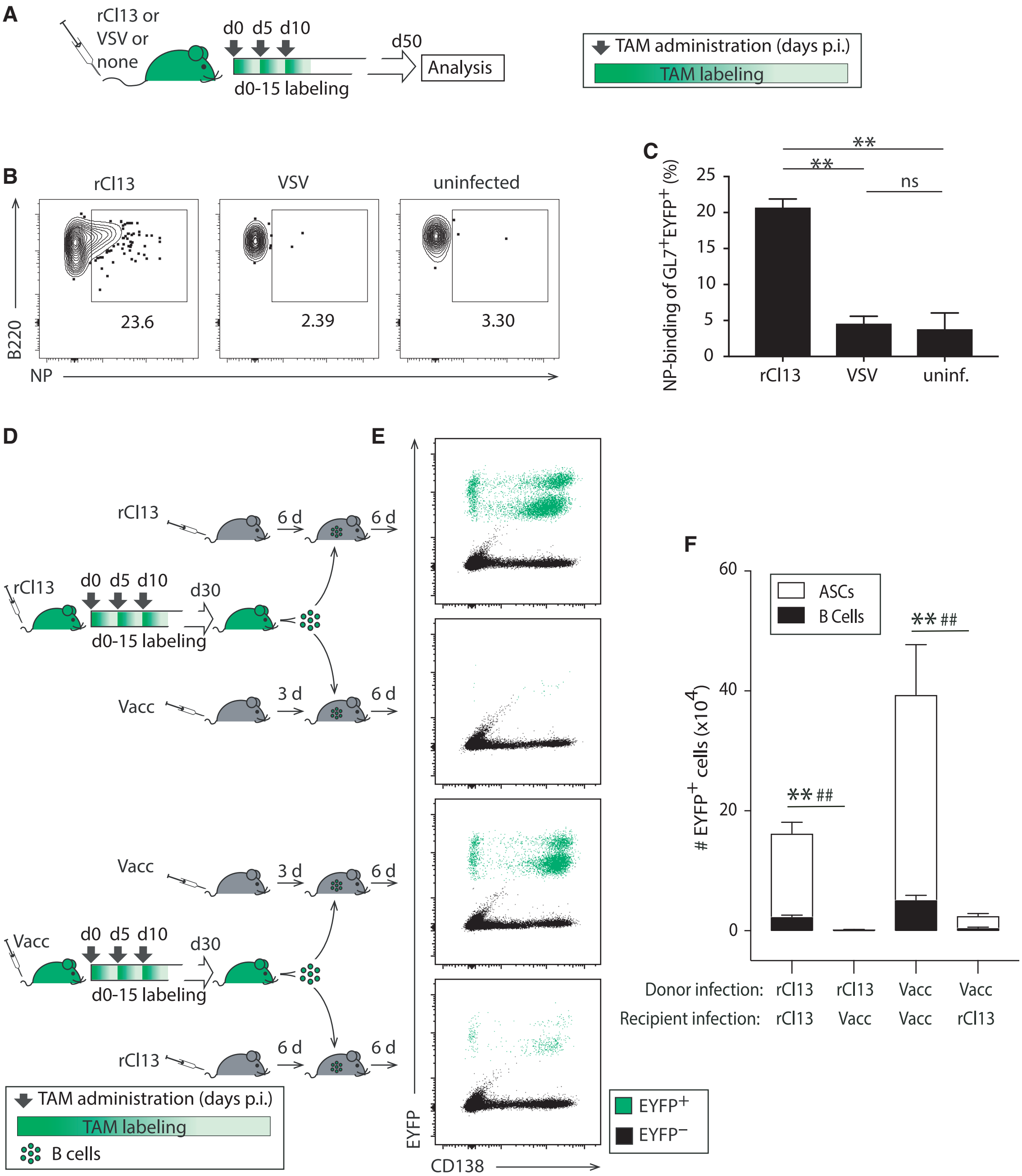

Figure 2. AID Reporter System Identifies LCMV-Specific B Cells in a Polyclonal Response

(A–C) We infected AID^{rep} mice with rCl13 or VSV on day 0 or left them uninfected. TAM was administered on days 0, 5, and 10, as schematically shown in (A). LCMV-NP binding by splenic EYFP^{+} GL7^{+} B cells was analyzed on day 50 (B and C). Panel (B) shows a representative FACS plot with numbers indicating the percentage of gated cells, as quantified in (C). For gating strategy, see Figure S2.

(D–F) We infected AID^{rep} mice with rCl13 or Vacc on day 0 and treated them with tamoxifen on days 0, 5, and 10, as schematically shown in (D). On day 30, we purified B cells from spleen by magnetic cell separation and transferred them into syngeneic C57BL/6 recipients, which had been infected with rCl13 6 days before (day 24) or with Vacc 3 days earlier (day 27). Six days after transfer (day 36), we measured the expansion and plasma cell differentiation of proliferated (CTV^{lo}) adoptively transferred EYFP-reporting B cells in the spleen (E and F). Two distinct EYFP^{+} ASC populations in (E) correspond to different stages of maturation. EYFP-reporting cells were enumerated in (F). Representative FACS plots are gated on EYFP^{+} B220^{+} CD138^{-} GL7^{+} B cells (B) and on recipient (CD45.2^{+}) lymphocytes in black with EYFP^{+} CTV^{lo} donor cells overlaid in green (E), respectively (see Figure S2). Numbers in FACS plots represent percentages of gated cells among EYFP^{+} B220^{+} CD138- GL7^{+} B cells.

Bars represent means ± SEM, n = 3–4 (C) and n = 4 (F). N = 2. One-way ANOVA with Bonferroni's post-test for multiple comparisons (C). Two-way ANOVA with Bonferroni's post-test for multiple comparisons (F). **p < 0.01 comparing B cells; $^{##}$p < 0.01 comparing ASCs, respectively. See also Figure S2.

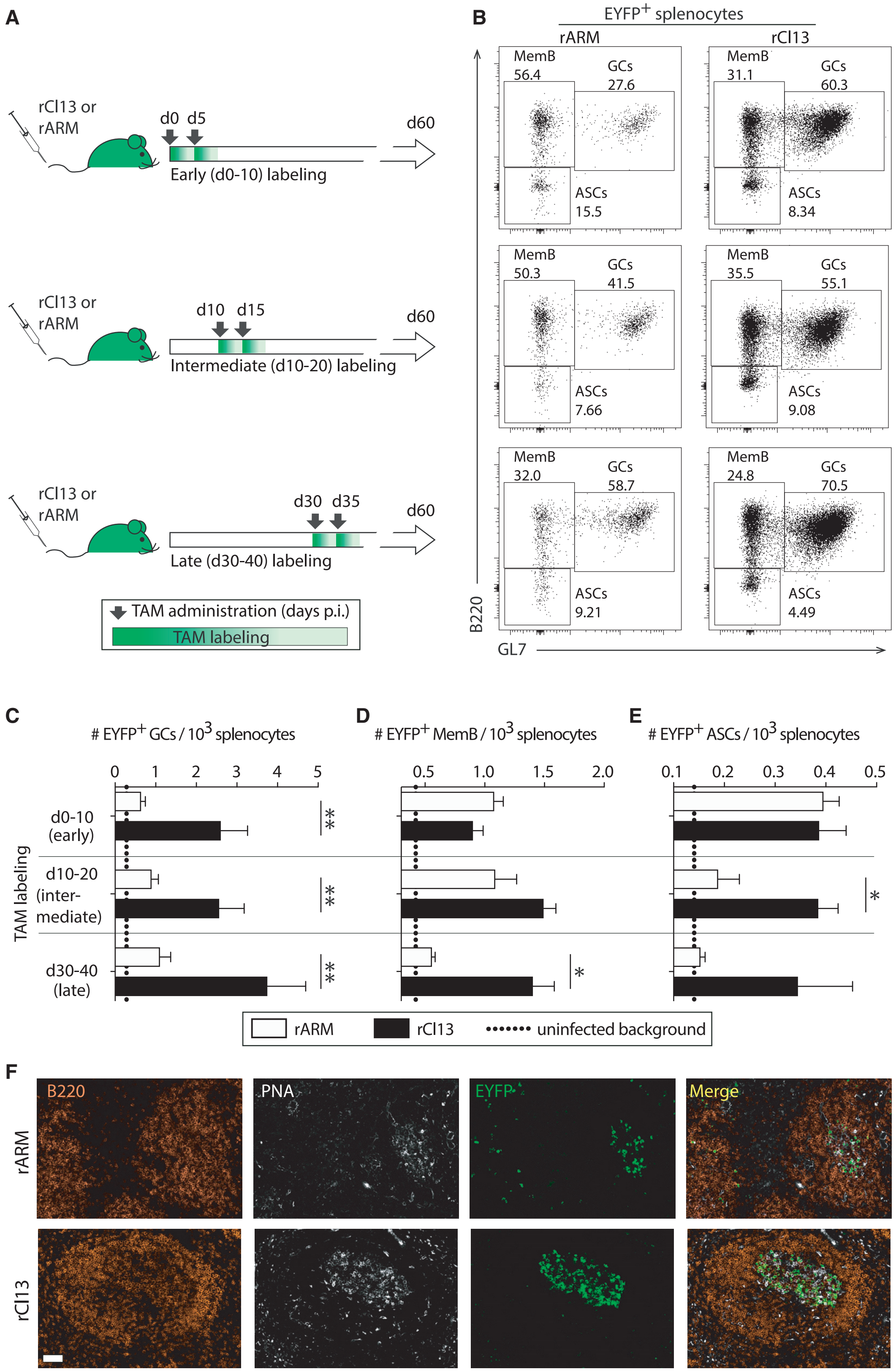

(legend on next page)

the KL25 antibody because of only intermediate affinity binding as opposed to high-affinity interaction with WE-GP (Hangartner et al., 2006). Intermediate affinity B cell receptor interactions were chosen for these experiments with KL25HL-AID^{rep} B cells to facilitate affinity maturation and render the experimental conditions more physiological. For induction of the AID-driven Cre reporter, the recipients were given TAM on the day of B cell transfer and again 3 days later (days 20 and 23). Within 5 days after adoptive transfer (day 25), virtually all KL25HL-AID^{rep} B cells had diluted CTV in both chronically and acutely infected mice (Figure S4), indicating antigen-driven proliferation (Schweier et al., 2019). The proportion of KL25HL-AID^{rep} cells, which had activated AID (EYFP$^+$) and/or expressed the GC marker GL7, increased from day 25 to day 35 in both rCl13*- and rARM*-infected recipients (Figures 4B and S4). In addition, the EYFP/GL7 subset distribution, i.e., the proportion of KL25HL-AID^{rep} B cells activating AID and participating in GC reactions, was similar in acutely and chronically infected mice. Importantly, however, the number of KL25HL-AID^{rep} B cell progeny recovered from chronically rCl13*-infected mice exceeded those emerging from rARM* infection by ~5-fold (Figure 4C). These numeric differences manifested in the AID-reporting (EYFP$^+$) and non-reporting (EYFP$^-$) compartments and were similarly prominent for GC B cells (GL7$^+$) as they were for MemB cells (GL7$^-$). Accordingly, KL25HL-AID^{rep} B cells produced substantially higher antibody titers when transferred into chronically infected mice than upon transfer into acutely infected animals (Figure 4D). This suggested that B cell clones recruited into the ongoing response or generated by hypermutation at later time points after infection were better represented in the serum immunoglobulin pool of chronically, rather than acutely, infected mice. Differential expansion of the adoptively transferred KL25HL-AID^{rep} B cell population was also evident at the histological level with markedly more EYFP$^+$ KL25HL-AID^{rep} B cells inside GCs as well as outside GCs (Figure 4E). In summary, these experiments established that, in the time window from days 20–35 after infection, virus-specific B cells expanded more vigorously in chronically, than in acutely, infected mice. They produced more antibody and yielded approximately 5-fold more AID-reporting GC B cell and MemB cell progeny.

Comparable V_H and J_H Intron Mutation Frequencies in B Cells of Acutely and Chronically Infected Mice

Next, we investigated whether chronic and acute infection settings differed in their ability to drive B cell hypermutation. We performed adoptive transfer experiments with KL25HL-AID^{rep} B cells on day 20 after infection and sorted GL7$^+$ GC B cell progeny on day 35, as outlined in Figure 4A. We determined both intronic sequences downstream of J_H as a readout of overall AID activity in the transferred KL25HL-AID^{rep} B cells and also KL25 V_H sequences as an indicator of affinity maturation. Neither of these target sequences provided clear evidence for a differential rate of hypermutation in acute, as compared with, chronic infection (Figures 5A and 5B). This conclusion found independent support when we compared J_H intronic sequences of polyclonal isotype-switched GC B cells (GL7$^+$ IgM$^-$ IgD$^-$ B220$^+$) on day 60 after rCl13 and rARM infection (Figures S5A and S5B). Hence, we performed a detailed analysis of coding mutations in the KL25 V_H region of KL25HL-AID^{rep} B cells emerging from acute and chronic infection. We found evidence for an enrichment of recurrent mutations both in complementarity determining regions (CDRs) and framework regions (FRs) of the BCR (Figure 5C). When systematically analyzing these sequences for evidence of affinity maturation, we noted three CDRH3 mutations (W104L, F106L, and Y108S), which were found in at least four of eight individual mice infected with either rARM* or rCl13* (bold red residues in Figure 5C). With the exception of F106L, these recurrent CDRH3 mutations were found in KL25HL-AID^{rep} B cells recovered from chronically and acutely infected animals. We recombinantly expressed the three KL25 point mutant antibodies with amino acid changes in CDRH3 (W104L, F106L, and Y108S) and assessed their ability to bind WE-GP* and to neutralize the corresponding WE-GP*-expressing virus rCl13*. KL25-W104L was the most prevalent mutant antibody (7 out of the 8 mice studied), and it bound considerably better to WE-GP* than to the parental KL25 antibody, whereas the latter was a better binder on WE-GP (Figure 5D). Importantly, KL25-W104L neutralized rCl13* more potently than KL25-WT did, altogether indicating that the W104L mutation was acquired because of an affinity maturation on the WE-GP* envelope protein of the infecting rCl13* virus (Figure 5E). Improved WE-GP* binding and rCl13* neutralization by KL25-W104L was acquired at the expense of a relative reduction in the antibody's capacity to bind and neutralize rCl13. Similar trends were also noted for the other two CDRH3 KL25 mutants (KL25-F106L and KL25-Y108S; Figures S5C–S5F). Taken together, these data suggested that KL25HL-AID^{rep} B cells underwent affinity maturation upon adoptive transfer into either rCl13* or rARM* infected mice, and that this process was at least equally efficient in chronic infection as it was in acute infection.

Potent Selection of Hypermutated B Cells in Chronically Infected Mice

We report here that the generation of LCMV nAbs depends on hypermutation (Figures 1D–1F) and that nAbs are preferentially

Figure 3. Chronic Infection Triggers a Sustained GC Response with Prolonged Plasma Cell and Memory B Cell Output

(A) We infected AID^{rep} mice with rARM or rCl13 on day 0, followed by TAM administration on day 0 and day 5 (early), day 10 and day 15 (intermediate), or day 30 and day 35 (late), respectively.

(B) EYFP-expressing cells were analyzed 2 months after infection. Representative FACS plots are gated on EYFP$^+$ cells (see Figure S3). Numbers in FACS plots indicate percentages of gated cells among EYFP$^+$ cells.

(C–E) EYFP-expressing GC B cells (C), MemB cells (D), and ASCs (E) in the spleen were enumerated. Dotted lines indicate background levels of EYFP-expressing cells in uninfected control mice.

(F) Representative histological spleen sections from rCl13- and ARM-infected mice in the early labeled group.

Magnification bar: 50 μm. Bars represent means ± SEM, n = 4–5 (C–E) and n = 6 from three datasets (F). N = 2. Two-way ANOVA with Bonferroni's post-test for multiple comparisons. *$p < 0.05$, **$p < 0.01$. See also Figure S3.

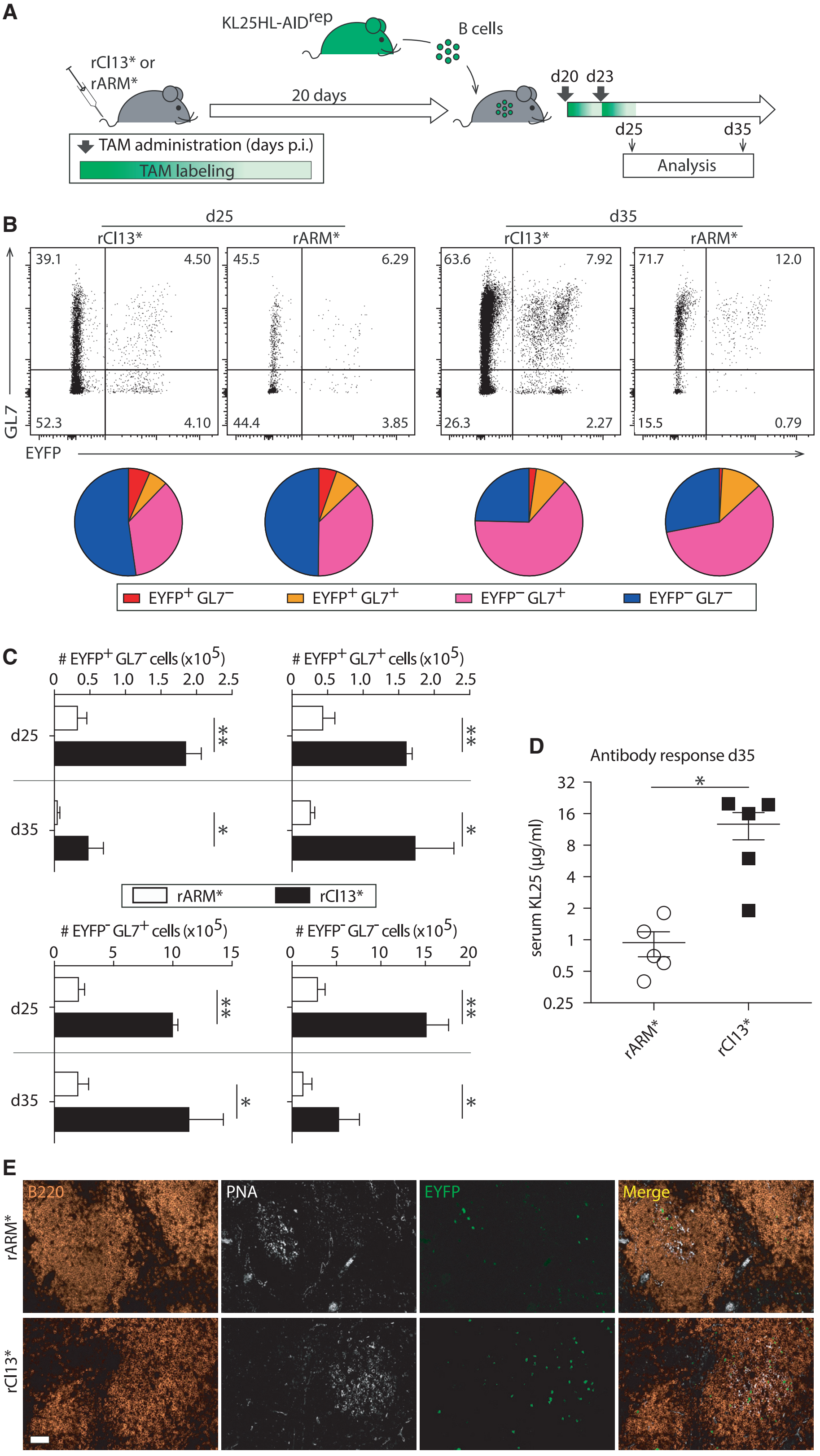

(legend on next page)

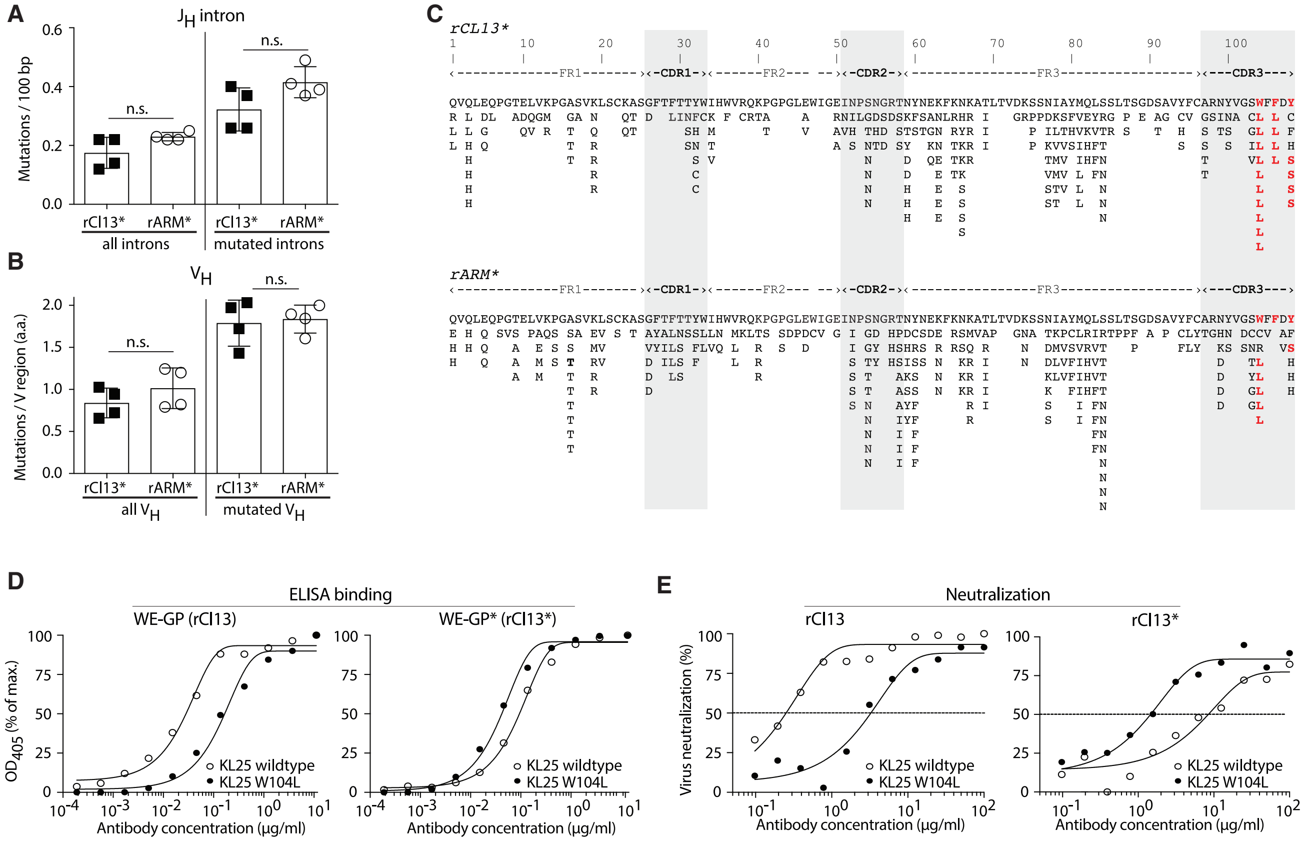

Figure 5. Comparable V_H and J_H Intron Mutation Frequencies in B Cells of Acutely and Chronically Infected Mice

(A–C) We performed adoptive transfer experiment as in Figure 4, and on day 36, we sorted isotype-switched adoptively transferred KL25HL-AID^{rep} GC B cells (CD45.1$^+$ CD45.2$^-$IgM$^-$IgD$^-$GL7$^+$B220$^+$) by FACS for IgH locus sequencing. (A) Mutation frequencies in the J_H intron as base pair (bp) changes are shown for all sequences obtained (left) as well as for mutated introns only (right). (B) Mutations in the KL25 V_H gene as total amino acid changes per V sequence, for all sequences (left) or sequences with amino acid changes (right), taking only functional sequences into account. (C) All amino acid mutations collected from four rARM*- and four rCl13*-infected mice (708 sequences total, 85–91 sequences per mouse) are represented along the KL25 V_H protein sequence (top: rCl13* infection; bottom: rARM* infection). Mutations are shown in bold red letters when present in at least four individual mice (out of eight). CDR positions are shaded in gray. Bars show means ± SD, and symbols represent individual mice. n = 4, N = 1 (A–C). Unpaired two-tailed Student's t test (A and B). ns, $p \geq 0.05$.

(D) Binding of KL25 wild-type and KL25-W104L to WE-GP and WE-GP*.

(E) Neutralization of rCl13 and rCl13* by KL25 wild-type and KL25-W104L. Symbols in (D) and (E) show the mean of 2 technical replicates. N = 3 (D and E). See also Figure S5.

mounted in chronic, as opposed to acute, infection (Figure 1C; Eschli et al., 2007). Moreover, we have previously shown that the formation of nAbs and also the control of chronic LCMV infection fail in *aicda*$^{-/-}$ mice lacking class-switch recombination and affinity maturation (Bergthaler et al., 2009). Hence, we aimed to test whether AID-driven hypermutation, which apparently functions efficiently in chronically infected mice (Figure 5), is rate limiting for nAb generation and virus control. As a model of hypermutation-impaired B cells, we made use of gene-targeted mice, which carry one deleted and one G23S-mutated allele of *aicda* (AID$^{G23S/-}$ mice [Wei et al., 2011]). The AID-G23S mutation allows for class switch recombination and causes a substantial

Figure 4. Chronic Infection Is a Potent Long-Term Driver of B Cell Responses

(A) We infected syngeneic recipient mice with rARM* or rCl13* on day 0, and 20 days later, we adoptively transferred CTV-labeled KL25HL-AID^{rep} B cells. TAM was administered on the day of transfer (day 20) and on day 23.

(B and C) Five and 15 days after adoptive transfer (day 25 and day 35), we analyzed KL25HL-AID^{rep} B cells by flow cytometry for AID reporting (EYFP$^+$) and GC differentiation (GL7 expression). Representative FACS plots in (B) are gated on adoptively transferred proliferated B cells (CD45.1$^+$ CD45.2$^-$ CTV^{lo} B220$^+$ CD138$^-$ cells, gating strategy in Figure S4). The average proportional representation of EYFP$^+$GL7$^-$ (red), EYFP$^+$GL7$^+$ (orange), EYFP$^-$GL7$^+$ (magenta), and EYFP$^-$GL7$^-$ (blue) subsets is displayed in pie charts. Absolute numbers of these same cell populations are shown in (C). Bars represent means ± SEM, n = 3–5.

(D) The concentration of KL25 IgG in the serum of KL25HL-AID^{rep} B cell recipients was determined by GP-1 ELISA. Background GP-1-specific IgG levels in control mice without KL25HL-AID^{rep} B cell transfer were at least 4-fold lower than in the respective groups of recipients. Symbols show individual mice.

(E) Histological sections from spleens of rCl13* and rARM* infected mice on day 25. Scale bar: 50 µm. n = 3, N ≥ 2.

Two-way ANOVA with Bonferroni's post-test for multiple comparisons (C), unpaired two-tailed Student's t test (D). *$p < 0.05$, **$p < 0.01$. See also Figure S4.

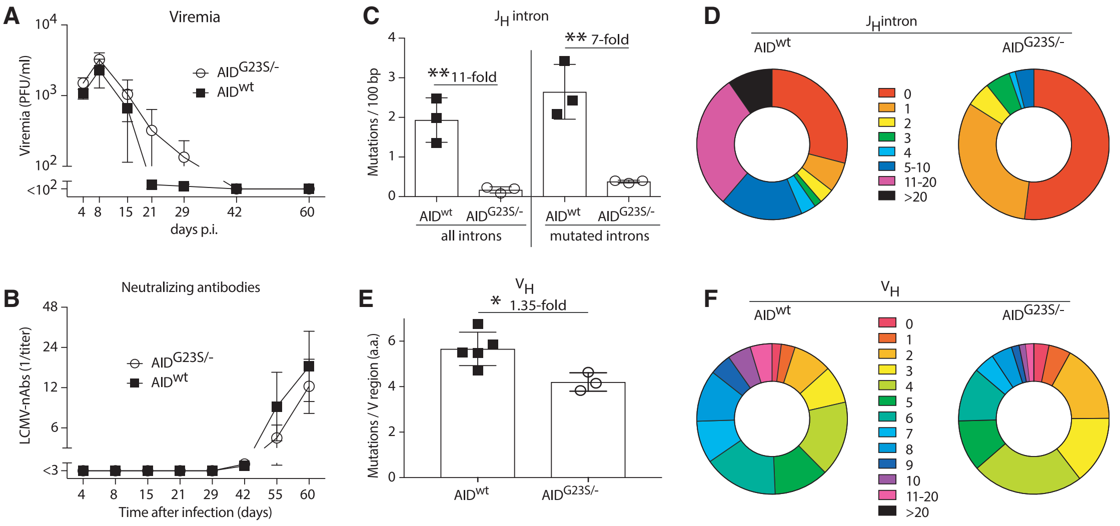

Figure 6. Potent Selection of Hypermutated B Cells in Chronically Infected Mice

(A and B) We infected $AID^{G23S/-}$ and WT control mice with rCl13 and measured viremia (A) and rCl13-neutralizing antibodies (B) over time.

(C and D) Sixty days after infection we sorted GC B cells ($GL7^{+}B220^{+}$) from the spleen for IgH locus sequencing. Number of nucleotide mutations in all J_H introns (left) and in mutated J_H introns (right, C). Distribution of J_H intron mutation numbers (mutations per individual sequence) in $AID^{G23S/-}$ and WT control mice (D). Results represent 74 and 62 sequences, respectively, from three mice per group.

(E and F) Number of amino acid mutations per sequenced V_H region (E) and distribution of V_H mutation numbers (F) in $AID^{G23S/-}$ and AID^{wt} control mice. 1.3×10^6 – 1.5×10^6 sequences per mouse from three to five mice per group were analyzed.

Symbols in (A) and (B) represent means ± SEM. Bars in (C) and (E) represent means ± SD, with symbols showing individual mice. Donut plots represent the distribution of sequences with the indicated mutation numbers. n = 7 to 8 (A and B), n = 3 (C and D). N = 2 (A, B, E, and F), N = 1 (C and D). $^{*}p < 0.05$, $^{**}p < 0.01$ by unpaired two-tailed Student's t test. The fold difference between groups is indicated (C and E).

reduction in hypermutation rates (Wei et al., 2011). We infected $AID^{G23S/-}$ and WT control mice with rCl13 to follow viremia and nAb kinetics. To our surprise, rCl13 control in $AID^{G23S/-}$ was only very modestly delayed, if at all (Figure 6A). In addition, nAb responses followed similar kinetics as in WT mice and reached comparable titers (Figure 6B). Hence, we sorted polyclonal $GL7^{+}$ GC B cells on day 60 after infection for Ig_H locus sequencing. The analysis of J_H intronic sequences as a readout of overall AID activity confirmed our expectations (Wei et al., 2011) of approximately 10-fold-reduced mutation frequencies in $AID^{G23S/-}$ mice (Figure 6C). This difference between $AID^{G23S/-}$ and WT mice persisted when excluding non-mutated sequences from the analysis ("mutated introns" in Figure 6C) and was also reflected in a paucity of highly mutated clones (Figure 6D). To understand how $AID^{G23S/-}$ mice generated LCMV nAbs despite substantially reduced hypermutation frequencies, we determined the mutational burden in polyclonal V_H sequences by unbiased high-throughput RNA sequencing (RNA-seq). In marked contrast to the intronic sequences, a very modest 1.3-fold reduction in the number of V_H mutations was observed in $AID^{G23S/-}$ mice (Figure 6E), with a fair representation of highly mutated clones (Figure 6F). It, therefore, appeared that the efficient selection of mutated clones enabled the formation of nAbs and the control of rCl13 infection even in hypermutation-impaired $AID^{G23S/-}$ mice. Taken together, these findings indicated that chronic infection provides a formidable selection machinery, which, even under conditions of substantially reduced AID-driven mutation rates, yields GC B cells with highly mutated V_H sequences.

DISCUSSION

Our study establishes that the humoral immune system and GC B cell responses, in particular, are very effective at coping with chronic viral antigen exposure. Long-lived GC B cell responses, efficient selection of hypermutated clones and high MemB cell, and ASC output altogether argue against humoral immune subversion, a conclusion which finds independent support in the accompanying paper (Kräutler, 2020 [this issue of *Cell Reports*]). Intriguingly, this response pattern reflects the opposite of CD8 T cell exhaustion, yet, from an evolutionary standpoint, it seems logical and beneficial for the host. Exhaustion is commonly thought of as nature's strategy to avoid fatal immunopathological consequences of inflammatory cytokine release and overshooting T cell cytotoxicity (Frebel et al., 2012). nAb-mediated virus clearance, in contrast, is an innocuous and virtually non-inflammatory process. Immune complex disease, the only known side effect of humoral immune defense in chronic viral infection, is rare and may selectively occur in a subset of patients with genetic predispositions (Gupta and Quigg, 2015).

It, therefore, appears that, with progression to the chronic phase of viral infection, the relative efficacy of humoral immune defense increases, whereas CD8 T cell responses undergo exhaustion. Interestingly, the CD4 differentiation profiles that predominate under these respective conditions are predicted to support a shift from cellular to humoral immune defense: with progression to chronicity T helper 1 (Th1) responses become exhausted (Brooks et al., 2005; Kaufmann et al., 2007; Oxenius et al., 1998), similar to $CD8^{+}$ T cells, whereas

T follicular helper (Tfh) cells start to dominate the CD4 T cell response (Fahey et al., 2011; Feng et al., 2012; Lindqvist et al., 2012). Chronic infection also favors the emergence of Tfh cells with distinct functional profiles, such as the ability to co-produce interleukin (IL)-10 and IL-21 (Xin et al., 2018). Accordingly, potent Tfh responses represent an essential component of efficient GC reactions in the chronic infection context (Greczmiel et al., 2017; Harker et al., 2011; Xin et al., 2018). Equally important, sustained high-level antigen supply for continuous B cell receptor signaling fosters potent GC B cell responses and likely is accountable for more sustained ASC output in rCl13-infected animals.

Our observations help understand why nAbs to LCMV in mice, in analogy to HIV and HCV in humans, are more readily elicited in chronic infection than upon acute infection or vaccination (Eschli et al., 2007; Kinchen et al., 2018; Law et al., 2013; Osburn et al., 2014; Pestka et al., 2007; Pinschewer et al., 2004; Raghuraman et al., 2012; Rose et al., 2000; Rusert et al., 2016; Sommerstein et al., 2015). One widely held concept relates to the longer evolutionary trajectory of B cell receptors, which result from prolonged antigenic stimulation and continuous affinity maturation (Burton and Hangartner, 2016). Our findings of comparable BCR hypermutation rates in chronically and acutely infected mice, although not contradictory to the above, suggest that additional mechanisms may be at work. (1) We propose that the continuously high ASC output even at late time points after the onset of chronic infection warrants a better representation of the hypermutated GC B cell repertoire in the circulating serum antibody pool. Conversely, the hypermutated B cell pool emerging from acute infection would merely be available for accelerated nAb formation upon re-infection (Gage et al., 2019; Schweier et al., 2019). The observation that not only rCl13-infected, but also a few rARM-infected, mice mounted nAb responses (Figure 1C, right panel), supports this concept. It demonstrates that B clones of neutralizing capacity can be generated in acute infection, as well. (2) A second independent element that we assume is favoring nAb formation in chronic infection consists of the elevated cellularity of the GC B cell response. Studies in HIV-infected children, as well as the search for minimally mutated HIV-bnAbs, suggest that neutralizing B cell clones may be the product of a few serendipitous, yet improbable, mutations, rather than of accumulated mutational load (Georgiev et al., 2014; Jardine et al., 2016; Simonich et al., 2016; Wiehe et al., 2018). Similarly, HCV-bnAbs can apparently be generated with a limited number of critical somatic mutations (Bailey et al., 2017). A large pool of randomly mutated and efficiently selected B cell clones is, therefore, most likely to readily yield a few potently neutralizing ones. In line with that mechanism, the accompanying paper describes the chronic infection that results in greater intra-clonal diversification of responding B cells than in acute infection. (3) The combination of multiple weakly neutralizing antibodies can synergistically create substantial neutralizing activity (Mankowski et al., 2018; Nowakowski et al., 2002; Zwick et al., 2001). Based on greater cellularity of the GC response and greater diversification (see above and accompanying paper), we expect chronic infection to yield a broader spectrum of virus-specific serum antibodies and, therefore, more synergy-based neutralizing activity than acute infection produces.

A limitation of our study consists in its focus on B cells, without a concomitant analysis of CD4 T cell responses. During the past years, however, a series of elegant studies have investigated the key contribution of Tfh cells to GC B cell responses in chronic LCMV infection, documenting also their effect on virus control (Greczmiel et al., 2017; Harker et al., 2011; Xin et al., 2018). Further, we acknowledge that our study focuses on the late stages of infection, i.e., from 3 weeks after infection onward. That choice was based on the late appearance of nAbs in rCl13-infected mice, with an intention to better understand the B cell correlate thereof. Accordingly, the present study complements previous reports from our laboratory and from others, which have investigated B cell responses at the onset of LCMV infection (Fallet et al., 2016; Moseman et al., 2016; Sammicheli et al., 2016). Finally, LCMV infection in mice represents a versatile model to investigate the effect of chronic antigen load on immune responses, whereas other features of human infections, such as the progressive loss of $CD4^+$ T cells in human HIV infection, are not recreated in the LCMV model but may also affect B cell responses.

In summary, our findings and those in the accompanying paper characterize GC B cell responses as an imperturbable element of antiviral defense, which operates effectively and even excels when continuously faced with high amounts of viral antigen. Thereby our study portrays the natural context in which GC B cell responses yield potent nAbs or even bnAbs against persisting viruses, depicting also the challenges yet to be met when attempting to safely mimic those processes for prophylactic vaccination.

STAR★METHODS

Detailed methods are provided in the online version of this paper and include the following:

- KEY RESOURCES TABLE
- LEAD CONTACT AND MATERIALS AVAILABILITY
- EXPERIMENTAL MODEL AND SUBJECT DETAILS
  - Mice and Ethics Statement
  - Viruses and Cell Lines
- METHOD DETAILS
  - Flow Cytometry and Cell Sorting
  - Adoptive B Cell Transfer, Tamoxifen, and Passive Antibody Administration
  - Immunohistochemistry and Image Analysis
  - Viruses, Virus Production, Titration, and Infection
  - Determination of Neutralizing Antibody Titers
  - Analysis of Somatic Mutations at the IgH Locus
  - High Throughput Sequencing
  - Enzyme-Linked Immunosorbent Assay (ELISA), Recombinant Proteins, and Monoclonal Antibodies
  - Monoclonal Antibodies
- QUANTIFICATION AND STATISTICAL ANALYSIS
  - ELISA Quantification and Curve Fitting
  - Statistical Analysis
- DATA AND CODE AVAILABILITY

SUPPLEMENTAL INFORMATION

Supplemental Information can be found online at https://doi.org/10.1016/j.celrep.2019.12.023.

ACKNOWLEDGMENTS

We wish to thank Kerstin Narr for helpful discussions and long-standing collaboration, Bénédicte Afonso for excellent technical assistance, Tatiana Fadeev for help with analysis of KL25 mutations, Karsten Stauffer for animal husbandry, and the DBM flow cytometry core facility for fluorescence-activated cell sorting (FACS). This work was supported by the European Research Council (ERC grants No. 310962 to D.D.P. and No. 693267 to C.-A.R.), by the Swiss National Science Foundation (project grants No. 310030_173132 to D.D.P and No. 310030_173010 to D.M., and project No. 323630_151472 to B.F.), by SystemsX.ch-AntibodyX RTD (to S.T.R.), and by the Fondation Princesse Grace de Monaco (to J.-C.W.).

AUTHOR CONTRIBUTIONS

B.F., Y.H., M.F., Y.I.E., V.G., U.M., D.M., S.T.R., J.-C.W., C.-A.R, and D.D.P. contributed to experimental conception and design; B.F., Y.H., M.F., K.C., A.V.d.l.A., G.G.Z., Y.I.E., V.G., U.M., K.H., D.M., S.T.R., J.-C.W., C.-A.R., and D.D.P. acquired, analyzed, and/or interpreted the data; B.F., Y.H., M.F., J.-C.W., C.-A.R., and D.D.P. drafted or critically revised the article for important intellectual content.

DECLARATION OF INTERESTS

D.D.P. is a founder, shareholder, and consultant and serves as chief scientific officer of Hookipa Pharma Inc., which is commercializing arenavirus-based vector technology. D.M. and D.D.P. are inventors on patents describing arenavirus-based vector technology. The remaining authors declare no competing interests.

Received: June 24, 2019
Revised: November 20, 2019
Accepted: December 6, 2019
Published: January 28, 2020

REFERENCES

Bailey, J.R., Flyak, A.I., Cohen, V.J., Li, H., Wasilewski, L.N., Snider, A.E., Wang, S., Learn, G.H., Kose, N., Loerinc, L., et al. (2017). Broadly neutralizing antibodies with few somatic mutations and hepatitis C virus clearance. JCI Insight *2*, 92872.

Battegay, M., Cooper, S., Althage, A., Bänziger, J., Hengartner, H., and Zinkernagel, R.M. (1991). Quantification of lymphocytic choriomeningitis virus with an immunological focus assay in 24- or 96-well plates. J. Virol. Methods *33*, 191–198.

Bergthaler, A., Merkler, D., Horvath, E., Bestmann, L., and Pinschewer, D.D. (2007). Contributions of the lymphocytic choriomeningitis virus glycoprotein and polymerase to strain-specific differences in murine liver pathogenicity. J. Gen. Virol. *88*, 592–603.

Bergthaler, A., Flatz, L., Verschoor, A., Hegazy, A.N., Holdener, M., Fink, K., Eschli, B., Merkler, D., Sommerstein, R., Horvath, E., et al. (2009). Impaired antibody response causes persistence of prototypic T cell-contained virus. PLoS Biol. *7*, e1000080.

Bergthaler, A., Flatz, L., Hegazy, A.N., Johnson, S., Horvath, E., Löhning, M., and Pinschewer, D.D. (2010). Viral replicative capacity is the primary determinant of lymphocytic choriomeningitis virus persistence and immunosuppression. Proc. Natl. Acad. Sci. USA *107*, 21641–21646.

Brooks, D.G., Teyton, L., Oldstone, M.B., and McGavern, D.B. (2005). Intrinsic functional dysregulation of CD4 T cells occurs rapidly following persistent viral infection. J. Virol. *79*, 10514–10527.

Bruns, M., Cihak, J., Müller, G., and Lehmann-Grube, F. (1983). Lymphocytic choriomeningitis virus. VI. Isolation of a glycoprotein mediating neutralization. Virology *130*, 247–251.

Burton, D.R., and Hangartner, L. (2016). Broadly neutralizing antibodies to HIV and their role in vaccine design. Annu. Rev. Immunol. *34*, 635–659.

Burton, A.R., Pallett, L.J., McCoy, L.E., Suveizdyte, K., Amin, O.E., Swadling, L., Alberts, E., Davidson, B.R., Kennedy, P.T., Gill, U.S., et al. (2018). Circulating and intrahepatic antiviral B cells are defective in hepatitis B. J. Clin. Invest. *128*, 4588–4603.

Charan, S., Hengartner, H., and Zinkernagel, R.M. (1987). Antibodies against the two serotypes of vesicular stomatitis virus measured by enzyme-linked immunosorbent assay: immunodominance of serotype-specific determinants and induction of asymmetrically cross-reactive antibodies. J. Virol. *61*, 2509–2514.

Chen, M., Sällberg, M., Sönnerborg, A., Weiland, O., Mattsson, L., Jin, L., Birkett, A., Peterson, D., and Milich, D.R. (1999). Limited humoral immunity in hepatitis C virus infection. Gastroenterology *116*, 135–143.

Di Niro, R., Lee, S.J., Vander Heiden, J.A., Elsner, R.A., Trivedi, N., Bannock, J.M., Gupta, N.T., Kleinstein, S.H., Vigneault, F., Gilbert, T.J., et al. (2015). *Salmonella* infection drives promiscuous B cell activation followed by extrafollicular affinity maturation. Immunity *43*, 120–131.

Dogan, I., Bertocci, B., Vilmont, V., Delbos, F., Mégret, J., Storck, S., Reynaud, C.A., and Weill, J.C. (2009). Multiple layers of B cell memory with different effector functions. Nat. Immunol. *10*, 1292–1299.

Doria-Rose, N.A., Schramm, C.A., Gorman, J., Moore, P.L., Bhiman, J.N., DeKosky, B.J., Ernandes, M.J., Georgiev, I.S., Kim, H.J., Pancera, M., et al.; NISC Comparative Sequencing Program (2014). Developmental pathway for potent V1V2-directed HIV-neutralizing antibodies. Nature *509*, 55–62.

Eschli, B., Zellweger, R.M., Wepf, A., Lang, K.S., Quirin, K., Weber, J., Zinkernagel, R.M., and Hengartner, H. (2007). Early antibodies specific for the neutralizing epitope on the receptor binding subunit of the lymphocytic choriomeningitis virus glycoprotein fail to neutralize the virus. J. Virol. *81*, 11650–11657.

Fahey, L.M., Wilson, E.B., Elsaesser, H., Fistonich, C.D., McGavern, D.B., and Brooks, D.G. (2011). Viral persistence redirects CD4 T cell differentiation toward T follicular helper cells. J. Exp. Med. *208*, 987–999.

Fallet, B., Narr, K., Ertuna, Y., Remy, M., Sommerstein, R., Cornille, K., Kreutzfeldt, M., Page, N., Zimmer, G., Geier, F., et al. (2016). Interferon-driven deletion of antiviral B cells at the onset of chronic infection. Sci. Immunol. *1*, eaah6817.

Feng, J., Hu, X., Guo, H., Sun, X., Wang, J., Xu, L., Jiang, Z., Xu, B., Niu, J., and Jiang, Y. (2012). Patients with chronic hepatitis C express a high percentage of $CD4^+CXCR5^+$ T follicular helper cells. J. Gastroenterol. *47*, 1048–1056.

Flatz, L., Bergthaler, A., de la Torre, J.C., and Pinschewer, D.D. (2006). Recovery of an arenavirus entirely from RNA polymerase I/II-driven cDNA. Proc. Natl. Acad. Sci. USA *103*, 4663–4668.

Frebel, H., Nindl, V., Schuepbach, R.A., Braunschweiler, T., Richter, K., Vogel, J., Wagner, C.A., Loffing-Cueni, D., Kurrer, M., Ludewig, B., and Oxenius, A. (2012). Programmed death 1 protects from fatal circulatory failure during systemic virus infection of mice. J. Exp. Med. *209*, 2485–2499.

Frey, S., Bertocci, B., Delbos, F., Quint, L., Weill, J.C., and Reynaud, C.A. (1998). Mismatch repair deficiency interferes with the accumulation of mutations in chronically stimulated B cells and not with the hypermutation process. Immunity *9*, 127–134.

Gage, E., Van Hoeven, N., Dubois Cauwelaert, N., Larsen, S.E., Erasmus, J., Orr, M.T., and Coler, R.N. (2019). Memory $CD4^+$ T cells enhance B-cell responses to drifting influenza immunization. Eur. J. Immunol. *49*, 266–276.

Gallimore, A., Glithero, A., Godkin, A., Tissot, A.C., Plückthun, A., Elliott, T., Hengartner, H., and Zinkernagel, R. (1998). Induction and exhaustion of lymphocytic choriomeningitis virus-specific cytotoxic T lymphocytes visualized using soluble tetrameric major histocompatibility complex class I-peptide complexes. J. Exp. Med. *187*, 1383–1393.

Georgiev, I.S., Rudicell, R.S., Saunders, K.O., Shi, W., Kirys, T., McKee, K., O'Dell, S., Chuang, G.Y., Yang, Z.Y., Ofek, G., et al. (2014). Antibodies VRC01 and 10E8 neutralize HIV-1 with high breadth and potency even with Ig-framework regions substantially reverted to germline. J. Immunol. *192*, 1100–1106.

Greczmiel, U., Kräutler, N.J., Pedrioli, A., Bartsch, I., Agnellini, P., Bedenikovic, G., Harker, J., Richter, K., and Oxenius, A. (2017). Sustained T follicular helper cell response is essential for control of chronic viral infection. Sci. Immunol. *2*, eaam8686.

Guidotti, L.G., Isogawa, M., and Chisari, F.V. (2015). Host-virus interactions in hepatitis B virus infection. Curr. Opin. Immunol. *36*, 61–66.

Gupta, A., and Quigg, R.J. (2015). Glomerular diseases associated with hepatitis B and C. Adv. Chronic Kidney Dis. *22*, 343–351.

Hangartner, L., Zellweger, R.M., Giobbi, M., Weber, J., Eschli, B., McCoy, K.D., Harris, N., Recher, M., Zinkernagel, R.M., and Hengartner, H. (2006). Nonneutralizing antibodies binding to the surface glycoprotein of lymphocytic choriomeningitis virus reduce early virus spread. J. Exp. Med. *203*, 2033–2042.

Harada, Y., Muramatsu, M., Shibata, T., Honjo, T., and Kuroda, K. (2003). Unmutated immunoglobulin M can protect mice from death by influenza virus infection. J. Exp. Med. *197*, 1779–1785.

Harker, J.A., Lewis, G.M., Mack, L., and Zuniga, E.I. (2011). Late interleukin-6 escalates T follicular helper cell responses and controls a chronic viral infection. Science *334*, 825–829.

Helle, F., Vieyres, G., Elkrief, L., Popescu, C.I., Wychowski, C., Descamps, V., Castelain, S., Roingeard, P., Duverlie, G., and Dubuisson, J. (2010). Role of N-linked glycans in the functions of hepatitis C virus envelope proteins incorporated into infectious virions. J. Virol. *84*, 11905–11915.

Jardine, J.G., Sok, D., Julien, J.P., Briney, B., Sarkar, A., Liang, C.H., Scherer, E.A., Henry Dunand, C.J., Adachi, Y., Diwanji, D., et al. (2016). Minimally mutated HIV-1 broadly neutralizing antibodies to guide reductionist vaccine design. PLoS Pathog. *12*, e1005815.

Kalinke, U., Bucher, E.M., Ernst, B., Oxenius, A., Roost, H.P., Geley, S., Kofler, R., Zinkernagel, R.M., and Hengartner, H. (1996). The role of somatic mutation in the generation of the protective humoral immune response against vesicular stomatitis virus. Immunity *5*, 639–652.

Kaufmann, D.E., Kavanagh, D.G., Pereyra, F., Zaunders, J.J., Mackey, E.W., Miura, T., Palmer, S., Brockman, M., Rathod, A., Piechocka-Trocha, A., et al. (2007). Upregulation of CTLA-4 by HIV-specific $CD4^+$ T cells correlates with disease progression and defines a reversible immune dysfunction. Nat. Immunol. *8*, 1246–1254.

Kinchen, V.J., Zahid, M.N., Flyak, A.I., Soliman, M.G., Learn, G.H., Wang, S., Davidson, E., Doranz, B.J., Ray, S.C., Cox, A.L., et al. (2018). Broadly neutralizing antibody mediated clearance of human hepatitis C virus infection. Cell Host Microbe. *24*, 717–730.e715.

Kräutler, et al. (2020). Quantitative and qualitative analysis of humoral immunity reveals continued and personalized evolution in chronic viral infection. Cell Reports *30*, 997–1012.

Krishnamurty, A.T., Thouvenel, C.D., Portugal, S., Keitany, G.J., Kim, K.S., Holder, A., Crompton, P.D., Rawlings, D.J., and Pepper, M. (2016). Somatically hypermutated plasmodium-specific IgM^+ memory B cells are rapid, plastic, early responders upon malaria rechallenge. Immunity *45*, 402–414.

Kwong, P.D., and Mascola, J.R. (2012). Human antibodies that neutralize HIV-1: identification, structures, and B cell ontogenies. Immunity *37*, 412–425.

Law, J.L., Chen, C., Wong, J., Hockman, D., Santer, D.M., Frey, S.E., Belshe, R.B., Wakita, T., Bukh, J., Jones, C.T., et al. (2013). A hepatitis C virus (HCV) vaccine comprising envelope glycoproteins gpE1/gpE2 derived from a single isolate elicits broad cross-genotype neutralizing antibodies in humans. PLoS ONE *8*, e59776.

Le Gallou, S., Zhou, Z., Thai, L.H., Fritzen, R., de Los Aires, A.V., Mégret, J., Yu, P., Kitamura, D., Bille, E., Tros, F., et al. (2018). A splenic IgM memory subset with antibacterial specificities is sustained from persistent mucosal responses. J. Exp. Med. *215*, 2035–2053.

Leist, T.P., Rüedi, E., and Zinkernagel, R.M. (1988). Virus-triggered immune suppression in mice caused by virus-specific cytotoxic T cells. J. Exp. Med. *167*, 1749–1754.

Lindqvist, M., van Lunzen, J., Soghoian, D.Z., Kuhl, B.D., Ranasinghe, S., Kranias, G., Flanders, M.D., Cutler, S., Yudanin, N., Muller, M.I., et al. (2012). Expansion of HIV-specific T follicular helper cells in chronic HIV infection. J. Clin. Invest. *122*, 3271–3280.

Malaspina, A., Moir, S., Orsega, S.M., Vasquez, J., Miller, N.J., Donoghue, E.T., Kottilil, S., Gezmu, M., Follmann, D., Vodeiko, G.M., et al. (2005). Compromised B cell responses to influenza vaccination in HIV-infected individuals. J. Infect. Dis. *191*, 1442–1450.

Mankowski, M.C., Kinchen, V.J., Wasilewski, L.N., Flyak, A.I., Ray, S.C., Crowe, J.E., Jr., and Bailey, J.R. (2018). Synergistic anti-HCV broadly neutralizing human monoclonal antibodies with independent mechanisms. Proc. Natl. Acad. Sci. USA *115*, E82–E91.

Menzel, U., Greiff, V., Khan, T.A., Haessler, U., Hellmann, I., Friedensohn, S., Cook, S.C., Pogson, M., and Reddy, S.T. (2014). Comprehensive evaluation and optimization of amplicon library preparation methods for high-throughput antibody sequencing. PLoS ONE *9*, e96727.

Moir, S., and Fauci, A.S. (2014). B-cell exhaustion in HIV infection: the role of immune activation. Curr. Opin. HIV AIDS *9*, 472–477.

Moir, S., Ho, J., Malaspina, A., Wang, W., DiPoto, A.C., O'Shea, M.A., Roby, G., Kottilil, S., Arthos, J., Proschan, M.A., et al. (2008). Evidence for HIV-associated B cell exhaustion in a dysfunctional memory B cell compartment in HIV-infected viremic individuals. J. Exp. Med. *205*, 1797–1805.

Moseman, E.A., Wu, T., de la Torre, J.C., Schwartzberg, P.L., and McGavern, D.B. (2016). Type I interferon suppresses virus-specific B cell responses by modulating $CD8^+$ T cell differentiation. Sci. Immunol. *1*, eaah3565.

Moskophidis, D., Laine, E., and Zinkernagel, R.M. (1993). Peripheral clonal deletion of antiviral memory $CD8^+$ T cells. Eur. J. Immunol. *23*, 3306–3311.

Nowakowski, A., Wang, C., Powers, D.B., Amersdorfer, P., Smith, T.J., Montgomery, V.A., Sheridan, R., Blake, R., Smith, L.A., and Marks, J.D. (2002). Potent neutralization of botulinum neurotoxin by recombinant oligoclonal antibody. Proc. Natl. Acad. Sci. USA *99*, 11346–11350.

Osburn, W.O., Snider, A.E., Wells, B.L., Latanich, R., Bailey, J.R., Thomas, D.L., Cox, A.L., and Ray, S.C. (2014). Clearance of hepatitis C infection is associated with the early appearance of broad neutralizing antibody responses. Hepatology *59*, 2140–2151.

Oxenius, A., Zinkernagel, R.M., and Hengartner, H. (1998). Comparison of activation versus induction of unresponsiveness of virus-specific $CD4^+$ and $CD8^+$ T cells upon acute versus persistent viral infection. Immunity *9*, 449–457.

Pestka, J.M., Zeisel, M.B., Bläser, E., Schürmann, P., Bartosch, B., Cosset, F.L., Patel, A.H., Meisel, H., Baumert, J., Viazov, S., et al. (2007). Rapid induction of virus-neutralizing antibodies and viral clearance in a single-source outbreak of hepatitis C. Proc. Natl. Acad. Sci. USA *104*, 6025–6030.

Pinschewer, D.D., Ochsenbein, A.F., Satterthwaite, A.B., Witte, O.N., Hengartner, H., and Zinkernagel, R.M. (1999). A Btk transgene restores the antiviral TI-2 antibody responses of xid mice in a dose-dependent fashion. Eur. J. Immunol. *29*, 2981–2987.

Pinschewer, D.D., Perez, M., Jeetendra, E., Bächi, T., Horvath, E., Hengartner, H., Whitt, M.A., de la Torre, J.C., and Zinkernagel, R.M. (2004). Kinetics of protective antibodies are determined by the viral surface antigen. J. Clin. Invest. *114*, 988–993.

Raghuraman, S., Park, H., Osburn, W.O., Winkelstein, E., Edlin, B.R., and Rehermann, B. (2012). Spontaneous clearance of chronic hepatitis C virus infection is associated with appearance of neutralizing antibodies and reversal of T-cell exhaustion. J. Infect. Dis. *205*, 763–771.

Richman, D.D., Wrin, T., Little, S.J., and Petropoulos, C.J. (2003). Rapid evolution of the neutralizing antibody response to HIV type 1 infection. Proc. Natl. Acad. Sci. USA *100*, 4144–4149.

Rose, N.F., Roberts, A., Buonocore, L., and Rose, J.K. (2000). Glycoprotein exchange vectors based on vesicular stomatitis virus allow effective boosting

and generation of neutralizing antibodies to a primary isolate of human immunodeficiency virus type 1. J. Virol. *74*, 10903–10910.

Rusert, P., Kouyos, R.D., Kadelka, C., Ebner, H., Schanz, M., Huber, M., Braun, D.L., Hozé, N., Scherrer, A., Magnus, C., et al.; Swiss HIV Cohort Study (2016). Determinants of HIV-1 broadly neutralizing antibody induction. Nat. Med. *22*, 1260–1267.

Salimzadeh, L., Le Bert, N., Dutertre, C.A., Gill, U.S., Newell, E.W., Frey, C., Hung, M., Novikov, N., Fletcher, S., Kennedy, P.T., and Bertoletti, A. (2018). PD-1 blockade partially recovers dysfunctional virus-specific B cells in chronic hepatitis B infection. J. Clin. Invest. *128*, 4573–4587.

Sammicheli, S., Kuka, M., Di Lucia, P., de Oya, N.J., De Giovanni, M., Fioravanti, J., Cristofani, C., Maganuco, C.G., Fallet, B., Ganzer, L., et al. (2016). Inflammatory monocytes hinder antiviral B cell responses. Sci. Immunol. *1*, eaah6789.

Schweier, O., Aichele, U., Marx, A.F., Straub, T., Verbeek, J.S., Pinschewer, D.D., and Pircher, H. (2019). Residual LCMV antigen in transiently $CD4^+$ T cell-depleted mice induces high levels of virus-specific antibodies but only limited B-cell memory. Eur. J. Immunol. *49*, 626–637.

Seiler, P., Bründler, M.A., Zimmermann, C., Weibel, D., Bruns, M., Hengartner, H., and Zinkernagel, R.M. (1998). Induction of protective cytotoxic T cell responses in the presence of high titers of virus-neutralizing antibodies: implications for passive and active immunization. J. Exp. Med. *187*, 649–654.

Simonich, C.A., Williams, K.L., Verkerke, H.P., Williams, J.A., Nduati, R., Lee, K.K., and Overbaugh, J. (2016). HIV-1 neutralizing antibodies with limited hypermutation from an infant. Cell *166*, 77–87.

Sommerstein, R., Flatz, L., Remy, M.M., Malinge, P., Magistrelli, G., Fischer, N., Sahin, M., Bergthaler, A., Igonet, S., Ter Meulen, J., et al. (2015). Arenavirus glycan shield promotes neutralizing antibody evasion and protracted infection. PLoS Pathog. *11*, e1005276.

Thai, L.H., Le Gallou, S., Robbins, A., Crickx, E., Fadeev, T., Zhou, Z., Cagnard, N., Mégret, J., Bole, C., Weill, J.C., et al. (2018). BAFF and $CD4^+$ T cells are major survival factors for long-lived splenic plasma cells in a B-cell-depletion context. Blood *131*, 1545–1555.

Wei, X., Decker, J.M., Wang, S., Hui, H., Kappes, J.C., Wu, X., Salazar-Gonzalez, J.F., Salazar, M.G., Kilby, J.M., Saag, M.S., et al. (2003). Antibody neutralization and escape by HIV-1. Nature *422*, 307–312.

Wei, M., Shinkura, R., Doi, Y., Maruya, M., Fagarasan, S., and Honjo, T. (2011). Mice carrying a knock-in mutation of Aicda resulting in a defect in somatic hypermutation have impaired gut homeostasis and compromised mucosal defense. Nat. Immunol. *12*, 264–270.

Wheatley, A.K., Kristensen, A.B., Lay, W.N., and Kent, S.J. (2016). HIV-dependent depletion of influenza-specific memory B cells impacts B cell responsiveness to seasonal influenza immunisation. Sci. Rep. *6*, 26478.

WHO (2018a). Hepatitis B Key Facts.https://www.who.int/news-room/fact-sheets/detail/hepatitis-b.

WHO (2018b). Hepatitis C Key Facts.http://www.who.int/news-room/fact-sheets/detail/hepatitis-c.

WHO (2018c). HIV Key Facts.http://www.who.int/news-room/fact-sheets/detail/hiv-aids.

Wiehe, K., Bradley, T., Meyerhoff, R.R., Hart, C., Williams, W.B., Easterhoff, D., Faison, W.J., Kepler, T.B., Saunders, K.O., Alam, S.M., et al. (2018). Functional relevance of improbable antibody mutations for HIV broadly neutralizing antibody development. Cell Host Microbe. *23*, 759–765.e6.

Xiao, X., Chen, W., Feng, Y., Zhu, Z., Prabakaran, P., Wang, Y., Zhang, M.Y., Longo, N.S., and Dimitrov, D.S. (2009). Germline-like predecessors of broadly neutralizing antibodies lack measurable binding to HIV-1 envelope glycoproteins: implications for evasion of immune responses and design of vaccine immunogens. Biochem. Biophys. Res. Commun. *390*, 404–409.

Xin, G., Zander, R., Schauder, D.M., Chen, Y., Weinstein, J.S., Drobyski, W.R., Tarakanova, V., Craft, J., and Cui, W. (2018). Single-cell RNA sequencing unveils an IL-10-producing helper subset that sustains humoral immunity during persistent infection. Nat. Commun. *9*, 5037.

Zajac, A.J., Blattman, J.N., Murali-Krishna, K., Sourdive, D.J., Suresh, M., Altman, J.D., and Ahmed, R. (1998). Viral immune evasion due to persistence of activated T cells without effector function. J. Exp. Med. *188*, 2205–2213.

Zellweger, R.M., Hangartner, L., Weber, J., Zinkernagel, R.M., and Hengartner, H. (2006). Parameters governing exhaustion of rare T cell-independent neutralizing IgM-producing B cells after LCMV infection. Eur. J. Immunol. *36*, 3175–3185.

Zwick, M.B., Wang, M., Poignard, P., Stiegler, G., Katinger, H., Burton, D.R., and Parren, P.W. (2001). Neutralization synergy of human immunodeficiency virus type 1 primary isolates by cocktails of broadly neutralizing antibodies. J. Virol. *75*, 12198–12208.

STAR★METHODS

KEY RESOURCES TABLE

REAGENT or RESOURCE	SOURCE	IDENTIFIER
Antibodies		
VL4 Rat anti-LCMV-NP	Dr. D.D. Pinschewer (Battegay et al., 1991)	N/A
Goat anti-Rat IgG-HRP	Jackson Immunoresearch	Cat#112-035-003; RRID: AB_2338128
Goat anti-Human IgG, Fc gamma-specific	Jackson Immunoresearch	Cat#109-005-098; RRID: AB_2337541
Goat anti-mouse IgG-HRP	Jackson Immunoresearch	Cat#115-035-205; RRID: AB_2338513
WEN-1	Dr. D.D. Pinschewer (Eschli et al., 2007)	N/A
WEN-3	Dr. D.D. Pinschewer (Seiler et al.,1998)	N/A
WEN-1UA	This paper	N/A
WEN-3UA	This paper	N/A
MOPC-21	BioXcell	Cat#BE0083; RRID: AB_1107784
KL25	Dr. D.D. Pinschewer (Bruns et al.,1983)	N/A
KL25-W104L	This paper	N/A
KL25-F106L	This paper	N/A
KL25-Y108S	This paper	N/A
Anti-mouse CD138 BV605	Biolegend	Cat#142516; RRID: AB_2562337
Anti-mouse B220 PE	BD Biosciences	Cat#553089; RRID: AB_394619
Anti-mouse IgM PercP-e710	eBioscience	Cat#46-5790-82; RRID: AB_1834435
Anti-mouse IgD APCCy7	BioLegend	Cat#405716; RRID: AB_10662544
Anti-mouse GL-7 eF450	eBioscience	Cat#48-5902-82; RRID: AB_10870775
Anti-mouse B220 PECy7	BioLegend	Cat#103222; RRID: AB_313005
Anti-mouse GL-7 AF647	BioLegend	Cat#144606; RRID: AB_2562185
Anti-mouse CD95 PE	BD Biosciences	Cat#554258; RRID: AB_395330
Anti-mouse CD45.1 BV785	BioLegend	Cat#110743; RRID: AB_2563379
Anti-mouse CD45.2 AF700	BioLegend	Cat#109822; RRID: AB_493731
Anti-mouse CD95 PECy7	BD Biosciences	Cat#557653; RRID: AB_396768
Anti-mouse B220 AF700	BioLegend	Cat#103232; RRID: AB_493717
Ani-mouse CD45.1 PE	BD Biosciences	Cat#553776; RRID: AB_395044
Anti-mouse CD45.2 BV785	BioLegend	Cat#109839; RRID: AB_2562604
Anti-mouse B220 FITC	BD Bioscience	Cat#553088; RRID: AB_394618
Anti-mouse CD3 FITC	BD Bioscience	Cat#553088; RRID: AB_394594
Anti-mouse CD45.1 Biotin	BioLegend	Cat#110703; RRID: AB_313492
Biotinylated Peanut Agglutinin (PNA)	Vector Laboratories	Cat#B-1075; RRID:AB_2313597
**Anti-mouse CD38 PerCP/Cy5.5**	BioLegend	Cat#102721; RRID: AB_2563332
Polyclonal rat IgG	BioXcell	Cat#BE0094; RRID: AB_1107795
Anti-GFP Antibody	ICL	Cat#RGFP-45ALY-Z
Anti-mouse B220 Alexa Fluor 647	BioLegend	Cat#103226; RRID: AB_389330
PNA Alexa Fluor 488	ThermoFischer Scientific	Cat#L21409; RRID: AB_2315178
Anti-mouse CD16/CD32	BioXcell	Cat#BE0307; RRID: AB_2736987
Bacterial and Virus Strains		
LCMV rCl13	Dr. D.D. Pinschewer (Flatz et al.,2006)	N/A
LCMV rARM	Dr. D.D. Pinschewer	N/A

(Continued on next page)

Continued

REAGENT or RESOURCE	SOURCE	IDENTIFIER
LCMV rCl13*	Dr. D.D. Pinschewer (Hangartner et al.,2006)	N/A
LCMV rARM*	Dr. D.D. Pinschewer (Hangartner et al.,2006)	N/A
Vaccinia virus	Dr. D.D. Pinschewer (Pinschewer er al., 1999)	N/A
Vesicular stomatitis virus	Dr. D.D. Pinschewer (Charan et al., 1987)	N/A
Chemicals, Peptides, and Recombinant Proteins		
Heparin Na 25000 I.E./5 ml	B. Braun Medical	Cat#B01AB01
DAB	DAKO	Cat#K5001
ABTS	Thermo Scientific	Cat#34026
Collagenase D	Roche	Cat#11088858001
DNaseI	Calbiochem	Cat#260913
TRI reagent LS	Sigma-Aldrich	Cat#T3934-100ML
Tactin-XT	Iba	N/A
Tamoxifen-NOLVADEX	AstraZeneca	N/A
Clinoleic	Baxter	Cat#FDB9501
Tissue-freezing-medium	Leica Microsystems	Cat#14020108926
GP-1-Fc	Dr. D.D. Pinschewer (Eschli et al., 2007)	N/A
WE-GP-Fc	Dr. D.D. Pinschewer (Hangartner et al., 2006)	N/A
WE-GP*-Fc	Dr. D.D. Pinschewer (Hangartner et al., 2006)	N/A
WE-GP-StreptagII	Dr. D.D. Pinschewer (Sommerstein et al., 2015)	N/A
LCMV-NP	Dr. D.D. Pinschewer (Fallet et al.,2016)	N/A
Brilliant Violet 605 Streptavidin	BioLegend	Cat#405229; RRID:AB_2313597
Critical Commercial Assays		
Zombie UV fixable viability kit	BioLegend	Cat#423108
Zombie Yellow fixable viability kit	BioLegend	Cat#423103
Alexa Fluor 647 Antibody Labeling Kit	Thermo Fischer Scientific	Cat#A20186
CellTrace Violet Cell Proliferation kit	Invitrogen	Cat#C34557
Pan B Cell Isolation Kit	Miltenyi Biotec	Cat#130-095-813
AccuScript High-Fidelity Reverse Transcriptase	Agilent Technologies	Cat#200820
TOPO-TA cloning kit	Invitrogen	Cat#450641
SPRI select beads	Beckman Coulter	Cat#B23319
KAPA Library Quantification Kit	KAPA Biosystems	Cat#KK4824
High Sensitivity NGS Fragment Analysis Kit	Advanced Analytical	Cat#DNF-474-1000
Q5 Hot Start High-Fidelity DNA polymerase	NEB	Cat#M0493
Vector® TrueVIEW Autofluorescence Quenching Kit	Vector Laboratories	Cat#SP-8400
Anti-biotin MicroBeads	Miltenyi Biotec	Cat#130-090-485
Deposited Data		
High-throughput B cell receptor sequencing data	NCBI Bioproject	Acc#PRJNA579837
Experimental Models: Cell Lines		
Hamster: BHK-21	ECACC	Cat#85011433; RRID: CVCL_1915
Mouse: NIH 3T3	ATCC	Cat#CRL-1658; RRID::CVCL_0594

(*Continued on next page*)

Continued

REAGENT or RESOURCE	SOURCE	IDENTIFIER
Experimental Models: Organisms/Strains		
Mouse: C57BL/6	The Jackson laboratory	JAX: 000664
Mouse: AID-Cre-EYFP, Aicdatm1.1(cre/ERT2)Crey x Gt(ROSA)26Sortm1(EYFP)Cos	Dr. J.C. Weill (Dogan et al., 2009)	N/A
Mouse: KL25HL; B6-IgH-J < tm1(VDJ-KL25)Zbz x B6J-Tg(KL25L)Tac	Dr. D.D. Pinschewer (Fallet et al., 2016)	N/A
Mouse: KL25L; B6J-Tg(KL25L)Tac	Dr. D.D. Pinschewer (Fallet et al., 2016)	N/A
Mouse: KL25HL-AID^{rep}, Aicdatm1.1(cre/ERT2)Crey x Gt(ROSA)26Sortm1(EYFP)Cos x B6-IgH-J < tm1(VDJ-KL25)Zbz x B6J-Tg(KL25L)Tac	This Paper	N/A
Mouse: AID^{G23S}; B6(Cg)-Aicda < tm2.1Hon >	Dr. T.Honjo (Wei et al., 2011)	N/A
Mouse: AID$^{G23S/-}$; B6(Cg)-Aicda < tm2.1Hon > x Aicdatm1.1(cre/ERT2)Crey	Dr. T.Honjo (Wei et al., 2011)	N/A
Oligonucleotides		
VH1: GAGGACTCTGCRGTCTATTWC	This Paper	N/A
VH3: GAGGACACACCCACATATTAC	This Paper	N/A
VH5: GAGGACACRGCCATGTATTAC	This Paper	N/A
VH6: GAAGACACTGGAATTTATTAC	This Paper	N/A
VH7: GAGGACAGTGCCACTTATTAC	This Paper	N/A
VH9: ATGAGGACATGGCTACATATTTC	This Paper	N/A
JH4rev: CACCAGACCTCTCTAGACAGC	This Paper	N/A
JH4-nested: TGAGACCGAGGCTAGATGCC	This Paper	N/A
VHDJH2: 5′ primer: CTCTCCGCAGGTGTCCACTCC	This Paper	N/A
VHDJH2: 3′ primer: AGAAAGAGGTTGTAAGGACTCAC	This Paper	N/A
JH2-JH4 segment: 5′ primer: CTAGGCACCACTC TCACAGTC	This Paper	N/A
JH2-JH4 segment: 3′ primer: CACCAGACCTCTCT AGACAGC	This Paper	N/A
Recombinant DNA		
Mouse HC IgG1 expression plasmid	Dr. Shozo Izui	N/A
Mouse LC expression plasmid	Dr. Shozo Izui	N/A
Software and Algorithms		
GEN5	BioTek Instruments	RRID: SCR_017317
GraphPad Prism 7	GraphPad Software	RRID: SCR_002798
FlowJo	Tree Star	RRID: SCR_008520
Ilumina MiSeq System	Illumina MiSeq	RRID: SCR_016379
PANDAseq	Panda	RRID: SCR_002705
CodonCode aligner	CodonCode Corporation	N/A
**IMGT/LIGM-DB**	IMGT/LIGM-DB	RRID: SCR_006931
**IgBlast**	IgBlast	RRID: SCR_002873
Adobe Photoshop CS6	Adobe Photoshop	RRID: SCR_014199

LEAD CONTACT AND MATERIALS AVAILABILITY

Further information and requests for resources and reagents should be directed to and will be fulfilled by the Lead Contact, Daniel D. Pinschewer (Daniel.Pinschewer@unibas.ch).

Material transfer agreements with standard academic terms will be established to document reagent sharing.

EXPERIMENTAL MODEL AND SUBJECT DETAILS

Mice and Ethics Statement

C57BL/6J wild-type mice were purchased from Charles River, France, CD45.1-congenic C57BL/6J have been obtained from the Swiss Immunological Mouse Repository, (SwImMR). AID-Cre-EYFP (AID^{rep}) mice carrying a targeted tamoxifen- (TAM-) inducible Cre recombinase (Cre-ERT2) in the *aicda* locus in combination with a Cre-inducible EYFP reporter gene in the ROSA26 locus have been described (Dogan et al., 2009). KL25HL mice, which express the monoclonal LCMV envelope-specific B cell receptor KL25 from a heavy chain knock-in and a light chain transgene, and KL25L mice, which express the light chain of the monoclonal LCMV envelope-specific B cell receptor KL25 as a transgene have been described (Fallet et al., 2016). KL25HL-AID^{rep} mice have been obtained by crossing AID-Cre-EYFP mice with KL25HL mice. AID^{G23S} mice (carrying a targeted G23S mutation on the *aicda* locus) were generously provided by Dr. T. Honjo (Wei et al., 2011). F1 offspring resulting from the intercross of homozygous AID^{G23S} and homozygous AID-Cre-EYFP mice were used as hypermutation-impaired AID$^{G23S/-}$ mice for experiments. All mice were on a C57BL/6 background and were bred at the Laboratory Animal Science Center (LASC) of the University of Zurich, Switzerland, under specific pathogen-free (SPF) conditions. Experiments were performed at the University of Basel and at the University of Geneva, in accordance with the Swiss law for animal protection and with authorizations from the Veterinäramt Basel-Stadt and from the Direction Générale de la Santé, Domaine de l'Expérimentation Animale of the Canton of Geneva, respectively. Assignment to experimental groups was based on sex- and age-matching. Adult animals of both genders were used to reduce the number of animals bred for research purposes. Study sample sizes in mouse experiments were chosen based on experience in our labs with respect to group sizes readily revealing biologically significant differences in the experimental models used. The groups were neither randomized nor were experiments conducted in a blinded fashion.

Viruses and Cell Lines

The recombinant LCMV strain Cl13 (rCl13) and Armstrong (rARM) expressing the surface glycoprotein of the LCMV strain WE (WE-GP) and the WE-GP N121K variants (rCl13*, rARM*) have been described (Sommerstein et al., 2015) and were engineered by standard procedures (Flatz et al., 2006). Recombinant Vaccinia virus expressing VSVG and vesicular stomatitis virus serotype Indiana (VSV) have been described (Charan et al., 1987; Pinschewer et al., 1999). For virus production and titration BHK-21 cells (Clone 13, ECAAC), BSC40 cells (ATCC) and NIH 3T3 cells (ATCC) were used and were confirmed to be mycoplasma-negative. Owing to their origin from renowned international repositories they were not authenticated.

METHOD DETAILS

Flow Cytometry and Cell Sorting

Single cell suspensions were obtained from spleens by mechanical or enzymatic digestion. Spleens were harvested in PBS containing 5% FCS and for mechanical digestion spleens pushed through a metal mesh in a Petri dish and homogenized by pipetting. For enzymatic digestion spleens where cut into small pieces and incubated with collagenase D (Roche) and DNaseI (Calbiochem) at 37°C for one hour on magnetic rotators and then washed with PBS. Dead cells were excluded using the Zombie UV™ or Zombie Yellow™ Fixable Viability Kits (BioLegend), then washed with PBS. The following fluorophore-conjugated antibodies were used for staining: αB220 (BioLegend or BD Biosciences), αCD138 (BioLegend), GL7 (eBioscience or BioLegend), αCD95 (BD Biosciences), αCD38 (BioLegend), biotinylated PNA (Vector Laboratories) and fluorophore-conjugated streptavidin (BioLegend). Anti-CD16/32 antibody and polyclonal rat IgG were added to the staining mixture to block Fc receptors. When adoptive cell transfers were performed, donor and recipient cells were distinguished using αCD45.1- and αCD45.2 - fluorophore-conjugated antibodies (BioLegend). Cells were co-stained for 15 minutes at 4°C with combinations of the fluorophore- and biotin-conjugated antibodies and with fluorophore-conjugated streptavidin, then washed with FACS-Buffer. Subsequently, the cells were fixed using PBS containing 4% PFA. NP-binding cells were detected using a bacterially expressed, Alexa647-labeled recombinant NP (Fallet et al., 2016; Schweier et al., 2019). All media were adapted to mouse osmolarity. Labeled EYFP$^+$ (AID- reporting) cells (see section "mice") were detected using an LSRFortessa flow cytometer (Becton Dickinson). Data were analyzed using FlowJo software (Tree Star). Absolute splenocyte counts of ARM-infected and Cl13-infected mice were indistinguishable when compared at either five or eight weeks after infection.

For sorting of KL25HL-AID^{rep} B cell progeny, splenocytes were positively enriched for CD45.1$^+$ cells by magnetic-activated cell sorting (MACS) using biotin-coupled αCD45.1 antibody (BioLegend) and anti-Biotin MicroBeads (Miltenyi Biotec) following the provider's instructions. The cells were then stained with the following antibodies: αCD45.1 (BioLegend), αCD45.2 (BioLegend), αB220 (BioLegend) and GL7 (BioLegend). CD45.2$^-$CD45.1$^+$B220$^+$GL7$^+$ cells were sorted into PBS containing 5% FCS (1'000 to 10'000 cells). Dried pellets were frozen in liquid nitrogen and DNA was prepared by cell lysis (Frey et al., 1998).

For sorting of AID$^{G23S/-}$ GC B cells, 5'000 B220$^+$GL7$^+$ splenocytes from each mouse were sorted directly into TRI Reagent LS (Sigma-Aldrich). RNA was extracted as described below (see section "high throughput sequencing"). DNA was extracted using chloroform phase separation according to manufacturer's procedures (Sigma-Aldrich), yeast tRNA (Sigma-Aldrich) was added as a carrier.

Cell sorting was performed using a FACSAria II cell sorter (Becton Dickinson).

Adoptive B Cell Transfer, Tamoxifen, and Passive Antibody Administration

For adoptive transfer of CD45.1^{+} KL25HL-AID^{rep} B cells, CellTrace Violet (Invitrogen) -labeled bulk splenocytes (2 × 10^{6} cells per recipient) or purified B cells (2 × 10^{5} cells per recipient) were injected intravenously in balance salt solution. B cells were purified by magnetic activated cell sorting using the Pan B Cell Isolation Kit for untouched B cells (Miltenyi Biotec). Syngenic (CD45.2^{+}) C57BL/6J mice served as recipients for short-term transfers. Transfers of KL25HL cells for analysis >1 week later were performed in KL25L recipients to avoid rejection by anti-idiotypic responses (Fallet et al., 2016).

For adoptive transfer of Vaccinia virus- or LCMV-experienced EYFP^{+} B cells we infected AID^{rep} mice on day 0 with the respective viruses, followed by tamoxifen treatment on day 0, 5 and 10. On day 30 splenic B cells were MACS-purified (Miltenyi Biotec Pan B Cell Isolation Kit for untouched B cells) and labeled with CTV. 2x10^{6} – 8x10^{6} B cells (purity >95%) containing between 10^{5} – 1.5x10^{5} EYFP^{+} B cells were transferred into Vaccinia virus- or LCMV-infected CD45.1^{+}-congenic C57BL/6J recipients.

Tamoxifen (2.5 mg Novadex (AstraZeneca) in 20% Clinoleic (Baxter)) was administered by gavage.

Passive antibody administration was performed three days after infection by an intravenous bolus injection of 300 μg purified antibody in PBS.

Immunohistochemistry and Image Analysis

For the histological sections shown in Figure 3, tissues were fixed in PBS containing 4% paraformaldehyde (Merck) for at least 4 hours at 4°C, then washed with PBS and cryoprotected with PBS-30% sucrose (Sigma-Aldrich) for at least 2 hours at 4°C. Tissues were then embedded in Tissue-freezing-medium (Leica Microsystems) and frozen on dry ice. For the microsections shown on Figure 4, tissues were fixed in PBS containing 4% paraformaldehyde (Merck) for at least 4 hours at 4°C, then washed with PBS and embedded in paraffin. Immunostaining was performed on 3 μm-thick sections using antibodies against GFP (ICL lab), Alexa Fluor 647-directly labeled B220 (eBioscience) and Lectin PNA Alexa Fluor 488 Conjugate (ThermoFisher). Immunostained slides were incubated with the Vector® TrueVIEW Autofluorescence Quenching Kit to remove autofluorescence signal (Vector Laboratories). Stained sections were scanned using a Panoramic Digital Slide Scanner 250 FLASH II (3DHISTECH) at 200 x magnification. Contrast was adapted using the "levels," "curves," "brightness" and "contrast" tools in Photoshop CS6.

Viruses, Virus Production, Titration, and Infection

For LCMV batch production, BHK-21 cells (ATCC) were infected with the respective viruses at MOI = 0.01 and virus-containing supernatant was harvested 48 hours later.

Recombinant Vaccinia virus expressing VSVG was produced on BSC-40 cells and vesicular stomatitis virus serotype Indiana (VSV) was grown on BHK-21 cells (MOI = 0.01 for 24 hours) (Pinschewer et al., 1999).

LCMV infectivity in blood was determined by immunofocus assay (Battegay et al., 1991). In brief, blood was collected into BSS supplemented with 1 IE/ml heparin (Na-heparin, Braun). Ten-fold serial dilutions were performed and were mixed with NIH 3T3 cells (ATCC) in 24-well plates, followed by 3 hours of incubation at 37°C. Then, overlay (1% methylcellulose in DMEM) was added and the cultures were incubated for two days. On the third day, supernatant was removed, the cells were fixed with 4% paraformaldehyde and permeabilized (1% Triton X-100 in PBS). After blocking (5% FCS), infectious foci were visualized using VL4 rat-anti-LCMV-NP antibody and secondary HRP-conjugated goat-anti-rat-IgG (Jackson Immunoresearch), followed by a color reaction (DAB).

rARM and rARM* infections were performed by intraperitoneal (i.p.) injection of 200 plaque-forming units (PFU). rCl13 and rCl13* were administered intravenously (i.v.) at a dose of 2x10^{6} – 4x10^{6} PFU. Vaccinia virus and VSV were administrated i.v. at doses of 2x10^{6} and 2x10^{7} PFU, respectively.

Determination of Neutralizing Antibody Titers

Neutralizing antibodies were measured in standard immunofocus assay-based (Battegay et al., 1991) plaque reduction neutralization tests. Serial two-fold dilutions of antibody-containing sera and of purified monoclonal antibodies were conducted in MEM 2% FCS in a 96-well format. Approximately 50 PFU of virus was added to each well, followed by 90 minutes of incubation at 37°C. NIH 3T3 fibroblasts were added and incubated with the virus – antibody mixture at 37°C for 90 minutes, allowing the cells to adhere. Thereafter, overlay (1% methylcellulose in DMEM) was added. Two days later, infectious foci were identified as described for the LCMV immunofocus assay. Neutralizing titers in mouse sera were determined as the highest serum dilution yielding at least 50% focus formation reduction. For monoclonal antibodies, the number of infectious foci at a given antibody concentration was expressed as a percentage of the number of foci in control wells without antibody added.

Analysis of Somatic Mutations at the IgH Locus

The J_H4 intronic sequence flanking rearranged V_H gene segments was amplified by PCR from DNA of sorted B cell subsets, from 10,000 to 1,000 cells. The PCR primers used are: in 5′, a mixture of five FR3 primers amplifying most V_H gene families (V_H1,: GAG GACTCTGCRGTCTATTWC, V_H3: GAGGACACACCCACATATTAC, V_H5: GAGGACACRGCCATGTATTAC, V_H6: GAAGACACTGG AATTTATTAC, V_H7: GAGGACAGTGCCACTTATTAC, V_H9: ATGAGGACATGGCTACATATTTC, in a 6:1:3:1:1 ratio); in 3′, a J_H4 intronic primer ($J_H4$rev: CACCAGACCTCTCTAGACAGC), with a nested amplification performed in cases of low cell numbers (using J_H4-nested: TGAGACCGAGGCTAGATGCC and the same V_H primer set) (2 min at 98°C and 50 cycles of 15 s at 98°C, 30 s at 64°C

and 30 s at 72°C, with Phusion® DNA polymerase (New England Biolabs); or 30 cycles plus 25 additional cycles in cases of nested PCR). Mutations were determined within 448 base pairs of the J_H4 intron.

For the KL25 VDJ knock-in allele, two different PCR were performed: one encompassing the V_HDJ_H2 (V1-53) coding sequence, and the other one based on the flanking intronic sequence, consisting in a fused J_H2-J_H4 segment present in the knock-in construct, using the following conditions. V_HDJ_H2: 5′ primer, CTCTCCGCAGGTGTCCACTCC; 3′ primer, AGAAAGAGGTTGTAAGGACTCAC; J_H2-J_H4 segment: 5′ primer, CTAGGCACCACTCTCACAGTC; 3′ primer, CACCAGACCTCTCTAGACAGC (2 min at 98°C and 40 cycles of 15 s at 98°C, 30 s at 64°C and 30 s at 72°C). Mutations were determined for the 324 bp sequence of the V_H gene (including CDR3), and for 570 bp of the intronic sequence, immediately downstream of J_H2.

PCR products were cloned with the TOPO-TA cloning kit (Invitrogen) and sequences were determined with an ABI Prism 3130xl Genetic Analyzer. Mutations were analyzed with the help of the CodonCode Aligner software. Between 16 to 91 sequences per sample were determined for mutation frequency determination.

High Throughput Sequencing

Preparation of antibody libraries from rCl13-infected WT and AID$^{G23S/-}$ mice: Library preparation by RT-PCR was performed similar to the protocol described in Menzel et al. (2014). Total RNA was extracted (25 μL elution volume) using the TRIzol Plus RNA Purification Kit (Life Technologies) according to the manufacturer's protocol. First-strand cDNA was synthesized with AccuScript High-Fidelity Reverse Transcriptase (Agilent Technologies) using total RNA and Oligo(dT) primers (Thermo Scientific) following the manufacturer's instructions. PCR- amplification was performed with Q5 Hot Start High-Fidelity DNA polymerase (NEB) in 50 μL reaction volumes with adjusted cycle numbers. For the eight germinal center samples an IgG-specific reverse primer was used. PCR1 products were purified using SPRI select beads (Beckman Coulter) at a ratio of 0.8X (elution in 30 μL water). Purified PCR1 products were submitted to PCR2-amplification, which adds full-length Illumina adapters to the library. Final products were gel-purified from 1% agarose gels. All amplicon libraries were quantified by qPCR using the KAPA Library Quantification Kit (KAPA Biosystems). Based on qPCR results, samples were diluted to 7 nM concentrations and a pool was prepared with equal amounts of library. Last, the pool was checked on a Fragment Analyzer (DNF-486 High Sensitivity NGS Fragment Analysis Kit, Advanced Analytical), the exact concentration determined (Qubit 2.0 Fluorometer) and diluted to a final concentration of 4 nM.

Illumina sequencing and data preprocessing: Illumina MiSeq sequencing was performed using 2x300 bp reads. Forward and reverse reads were paired using PANDAseq (Version 2.7, [2]) with parameter −0 300. Full-length VDJ region annotation and somatic mutation enumeration of successfully paired sequences was performed using ImMunoGeneTics (IMGT)/HighV-QUEST [3, 4]. For downstream analyses, sequences were pre-processed and reads only retained based on the following criteria: (i) the IMGT-indicated "Functionality" of the sequencing was "productive"; (ii) CDR3s were of minimal length of 4 amino acids; (iii) CDR3 were present with a minimum abundance of 2. Nucleotide mutation numbers were based on IMGT. Number of reads in analyses shown ranged between 1.3 and 1.5x10^6 per individual across both C57BL/6 and AID$^{G23S/-}$ mice.

Enzyme-Linked Immunosorbent Assay (ELISA), Recombinant Proteins, and Monoclonal Antibodies

GP-1-binding antibodies in mouse serum were determined using recombinant HEK293 cell-derived GP-1-Fc fusion protein as a substrate (Eschli et al., 2007). Binding of KL25 and the affinity-matured mutants KL25-W104L, KL25-F106L and KL25-Y108S to WE-GP and WE-GP* was determined using a synthetic HEK293 cell-derived fusion protein consisting of the extracellular WE-GP and WE-GP* domains, respectively, fused to human Fc (WE-GP-Fc, WE-GP*-Fc, [Hangartner et al., 2006]). For the aforementioned ELISA assay formats, 96-well high binding plates (Greiner Bio-One) were coated with 0.7 μg/ml goat anti-human IgG Fcγ antibody (Jackson, 109-005-098) in coating buffer (Na_2CO_3 15mM, $NaHCO_3$ 35mM, pH9.6) at 4°C overnight. Then, the plates were blocked for 2 hours at room temperature with PBS / 0.05% Tween / 5% milk (also used as buffer in the subsequent steps). Subsequently, GP-1-Fc, WE-GP-Fc or WE-GP*-Fc was added and incubated for 1h, followed by three wash steps with PBS / 0.05% Tween (PBS-T). Serum samples or monoclonal antibodies were added in a serially diluted manner and were incubated for 1h at RT. Following three more PBS-T wash steps, goat anti-mouse IgG-HRP conjugate antibody (1:750, Jackson) was added. Excess secondary antibody was washed away and HRP activity was detected using ABTS as a chromogen (Pierce). OD_{405} was determined in an ELISA reader.

The GP-binding capacity of WEN-1, WEN-1UA, WEN-3 and WEN-3UA was determined using as an ELISA substrate a HEK293-derived extracellular WE-GP domain with a C-terminal StreptagII sequence (GP-Streptag [Sommerstein et al., 2015]). The ELISA assay was performed analogously to the above, except that GP-Streptag was captured by coating ELISA plates with 0.5ug/ml of Strep-TactinXT (Iba), and that the plates were blocked with 0.2% BSA in PBS.

Monoclonal Antibodies

The unmutated ancestor sequences (WEN-1^{UA}, WEN-3^{UA}) of the rCl13-neutralizing monoclonal antibodies WEN-1 and WEN-3 (Figure S1; [Eschli et al., 2007; Seiler et al., 1998]) were identified by IgBlast (IgBlast) sequence analysis. All mutations deviating from the closest germline V(D)J heavy (HC) and light chain (LC) sequences were reverted. The corresponding cDNAs were synthesized (Genscript) and introduced into HC and LC expression cassettes for recombinant expression in a mouse IgG1 format (provided by Dr. Shozo Izui, University of Geneva). The antibodies were produced by transient co-transfection of the HC and LC expression plasmids in CHO cells (Protein Expression Core Facility, PECF, of the Swiss Federal Technical Highschool, EPFL, Lausanne, Switzerland). The antibodies were purified on an ÄKTAprime plus purification system using Protein G columns (GE healthcare). After

24 hours of PBS dialysis, the purified antibodies were quantified by IgG ELISA. MOPC-21 (BioXcell) served as isotype control antibody. The KL25-W104L, KL25-F106L and KL25-Y108S antibodies as well as a matching KL25 wild-type control antibody (Bruns et al., 1983) were obtained by analogous procedures, except that a mouse IgG2a expression format was used for these experiments.

QUANTIFICATION AND STATISTICAL ANALYSIS

ELISA Quantification and Curve Fitting

For titer determination in mouse serum based on standard curve fit, GEN5 software (BioTek Instruments) was used. Binding and neutralization curves of monoclonal antibodies were fitted using GraphPad Prism software 7 (GraphPad Prism).

Statistical Analysis

The GraphPad Prism software version 7 (GraphPad Software) was used for all statistical analyses. Unpaired two-tailed Student's t tests were performed to compare one parameter between two groups. For comparison of one parameter between multiple groups one-way analysis of variance (ANOVA) was performed and for comparison of multiple parameters between two or more groups two-way ANOVA was used, both followed by Bonferroni's post-test for multiple comparison. For statistical analysis of absolute cell counts, values were log-converted to obtain a near-normal distribution. *P values* ≥ 0.05 were regarded as not statistically significant (ns), p values < 0.05 as significant (*, #) and $p < 0.01$ as highly significant (**, ##).

The number of experimental animals "*n*" per group, number of experimental repeats "N," the type of error bar displayed and the tests performed for statistical analysis are indicated in each figure legend.

DATA AND CODE AVAILABILITY

High-throughput IgH sequencing data have been archived with the NCBI under BioProject number PRJNA579837. Additional raw datasets will be provided by the authors upon request.

Cell Reports, Volume *30*

Supplemental Information

Chronic Viral Infection Promotes Efficient Germinal Center B Cell Responses

Bénédict Fallet, Yi Hao, Marianna Florova, Karen Cornille, Alba Verge de los Aires, Giulia Girelli Zubani, Yusuf I. Ertuna, Victor Greiff, Ulrike Menzel, Karim Hammad, Doron Merkler, Sai T. Reddy, Jean-Claude Weill, Claude-Agnès Reynaud, and Daniel D. Pinschewer

Supplementary figure 1

```
WEN-1 HC
1        10        20        30        40        50        60        70        80        90        100       110       120       130
|        |         |         |         |         |         |         |         |         |         |         |         |         |
<-----------FR1---------><-CDR1-><------FR2------><-CDR2-><----------------FR3-----------------><----------CDR3---------><---JH---->
                              S                                 K          S    M                                   AM Y
QVQLQQSGPELVKPGASVKISCKASGYAFSNSWMNWVKQRPGKGLEWIGRIYPGDGDTNYNGKFRGKATLTADKSFSTAYIQLSSLTSEDSAVYFCARNIVGEGLYYDYDGGELFDYTVDCWGQGTSVTVSS

WEN-1 LC
1        10        20        30        40        50        60        70        80        90        100       110
|        |         |         |         |         |   CDR   |         |         |         |         |         |
<------------FR1---------><--CDR1--><------FR2------><2><--------------FR3-----------------><--CDR3-><---JK--->
                             D   I   N               A                           M            S           K
DIVLTQSPASLAVSLGQRATISCRASESVYNYGFSFMSWFQQKPGQPPKLLIYVASNQGSGVPARFSGSGSGTDFSLNIHPVEEDDTAMYFCQQTKEVPFTFGSGTNLEIK


WEN-3 HC
1        10        20        30        40        50        60        70        80        90        100       110       120
|        |         |         |         |         |         |         |         |         |         |         |         |
<-----------FR1---------><-CDR1-><------FR2------><-CDR2-><----------------FR3-----------------><----CDR3----><---JH---->
                       A  YT    Y N     P            GS NT    K     T   E   S   M     T
QVQLKQSGAELVRPGASVKLSCKTSGFIFTDYWIHWVKQRSGQGLEWIARIYPRTGTAYYNENFKGKAVLTADKSSNTAYLQLSSLKSEDSAVYFCARWRWPREVYNMDYWGQGTSVTVSS

WEN-3 LC
1        10        20        30        40        50        60        70        80        90        100       110
|        |         |         |         |         |   CDR   |         |         |         |         |         |
<------------FR1---------><--CDR1--><------FR2------><2><--------------FR3-----------------><--CDR3-><---JK--->
                          E  D YG   M      P                                    P              N             I
DIVLTQSPASLAVSLGQRATISCRASKSVASSDNSFLHWYQQKLGQPPKLLIYRASNLESGIPARFSGSGSRTDFTLTINSVEADDVATYYCQQSYEDPFTFGSGTKLELK
```

Supplementary Figure 1 (related to Figure 1): V(D)J amino acid sequences of the LCMV-neutralizing monoclonal antibodies WEN-1 and WEN-3 in comparison to their respective unmutated ancestors

Heavy and light chain (HC, LC) sequences are represented, with changes from their V_H/J_H and $V\kappa/J\kappa$ germline counterparts indicated above the sequence. CDR regions (IMGT nomenclature) are indicated in bold. The WEN-1 HC (IGHV1-82/D2-4/JH4) displays 8 nucleotide (and 7 amino acid) changes from germline with an unusual 25 amino acid-long CDR3; the WEN-3 HC (IGHV1-76/D2-3/JH4) has 33 nucleotide exchanges and 16 amino acid mutations. The WEN-1 LC (IGKV3-2/IGKJ4) harbors 12 nucleotide and 7 amino acid changes, and the WEN-3 LC (IGKV3-5/IGKJ4) comprises 13 nucleotide and 9 amino acid changes.

Supplementary figure 2

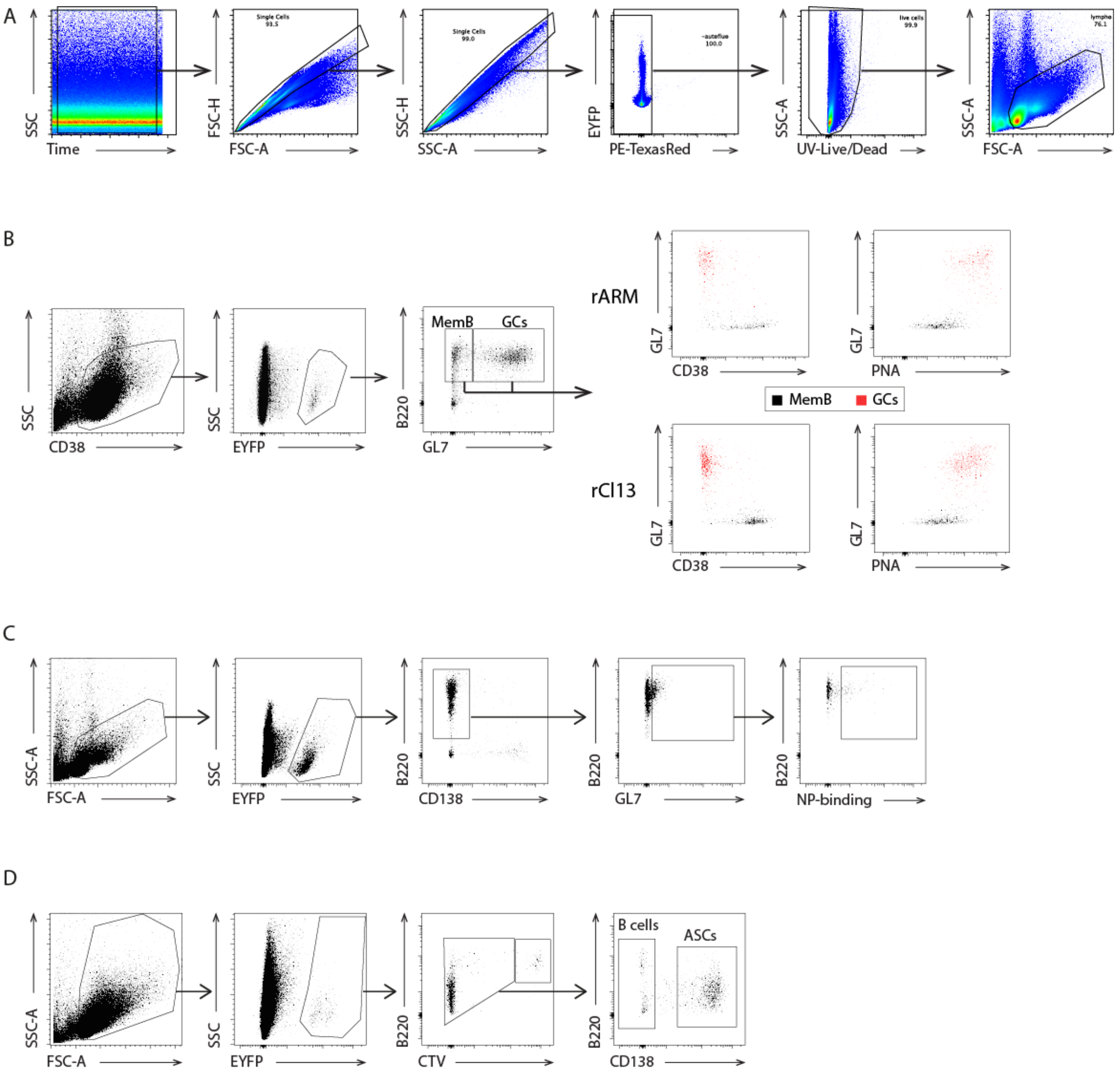

Supplementary Figure 2 (related to Figure 2): General gating strategy for viable lymphocytes, characterization of EYFP-labeled $GL7^+B220^+$ GC B cells and $GL7^-B220^+$ memory B cells, and gating strategy for the FACS analyses described in Figure 2

A. For all experiments in this manuscript lymphocytes were gated as described here: The first and last few events were gated out in a Time vs SSC gate. Doublets were then excluded based on FSC-A vs FSC-H and SSC-A vs SSC-H profiles. The PE-TexasRed channel was kept free and was used to exclude autofluorescent cells. Dead cells were stained as described in Methods and were gated out as shown. Lymphocytes were then gated based on forward- and side-scatter. All gating strategies in subsequent Supplementary figures and panels are based upon the analogous pre-gating strategy.

B: We infected AID^{rep} mice either rARM or rCl13 on day 0 and treated them with tamoxifen on day 0 and day 5. On day 35 we characterized PNA and CD38 profiles of EYFP-expressing $B220^+GL7^+$ GC B cells and of $EYFP^+$ $B220^+GL7^-$ MemB cells. Representative FACS plots from one out of four mice are shown except for GL7/CD38 and GL7/PNA plots, which display the combined events from two representative out of four mice.

C. We infected AID^{rep} mice with rCl13 or VSV on day 0 or left them untreated. Tamoxifen was administered on day 0, 5 and 10 as described for Fig. 2A. The gating strategy as shown was applied to quantify NP-binding cells.
D. We transferred Vacc-experienced or rCl13-experienced B cells into syngeneic C57BL/6 recipients, which had been infected with rCl13 six days before or with Vacc three days before, respectively, as described in Fig. 2D. The gating strategy as shown was used to enumerate proliferated CTV^{lo} EYFP^{+} B cells and ASCs.
Representative FACS plots are shown.

Supplementary figure 3

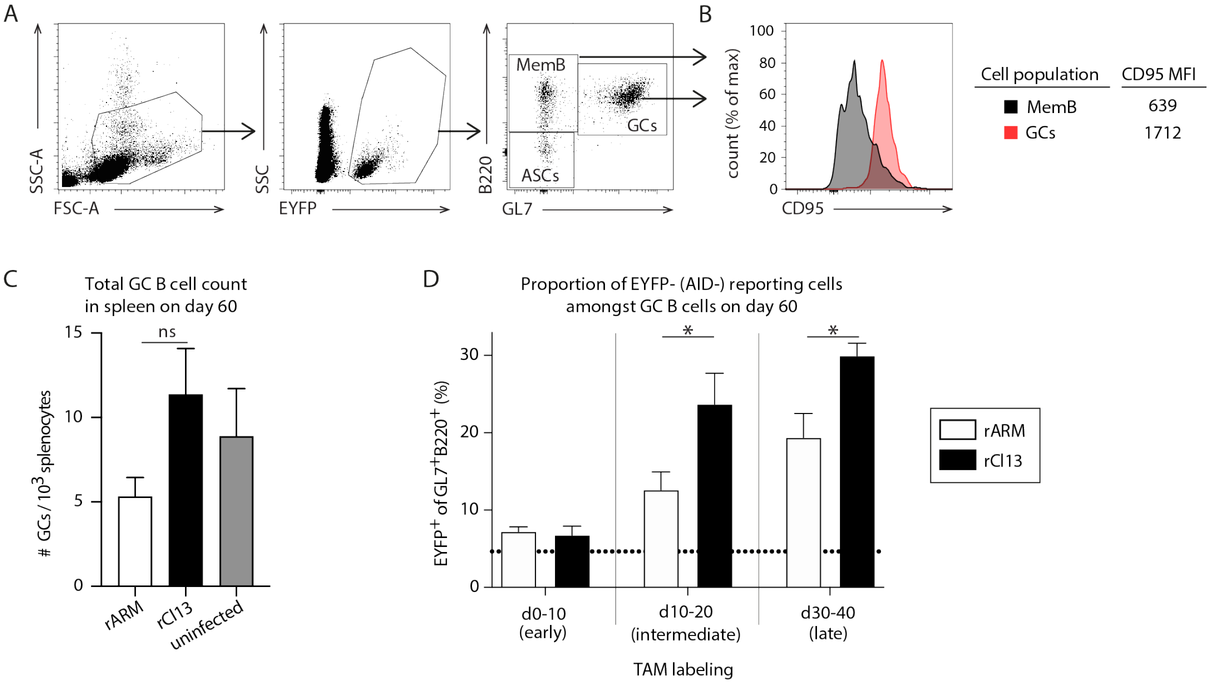

Supplementary Figure 3 (related to Figure 3): Gating strategy for the FACS analyses shown in Figure 3, total GC B cell counts and representation of EYFP-reporting GC B cells in spleens of rARM- and rCl13-infected mice on day 60.

A,B: We infected AID^{rep} mice with rARM or rCl13, followed by tamoxifen labeling on day 0 and 5 (early), 10 and 15 (intermediate) or 30 and 35 (late) after infection as described in Fig 3A. The gating strategy as displayed in (A) was used to quantify EYFP$^+$ GC B cells, MemB cells and ASCs on day 60 after infection. The CD95 expression profile of EYFP-expressing B220$^+$GL7$^+$ cells in comparison to EYFP$^+$ B220$^+$GL7$^-$ MemB cells was analyzed in (B). Representative FACS plots are shown.

C: We infected mice with rCl13 or rARM and left controls uninfected. Total GL7$^+$B220$^+$ GC B cells were enumerated in spleen 60 days later. One-way ANOVA with Bonferroni's post-test for multiple comparisons. ns: $p > 0.05$.

D: EYFP$^+$ (AID-reporting) GL7$^+$B220$^+$ GC B cells from Fig. 3C are displayed as a proportion of the total GL7$^+$B220$^+$ GC B cell compartment. Bars in (C,D) represent means +/- SEM. The dotted line indicates background EYFP reporting cells in uninfected control mice. n = 4 to 5, N = 2. Two-way ANOVA with Bonferroni's post-test for multiple comparisons. *: $p < 0.05$. Data in (C,D) are from the same experiment as reported in the main Fig. 3C.

Supplementary figure 4

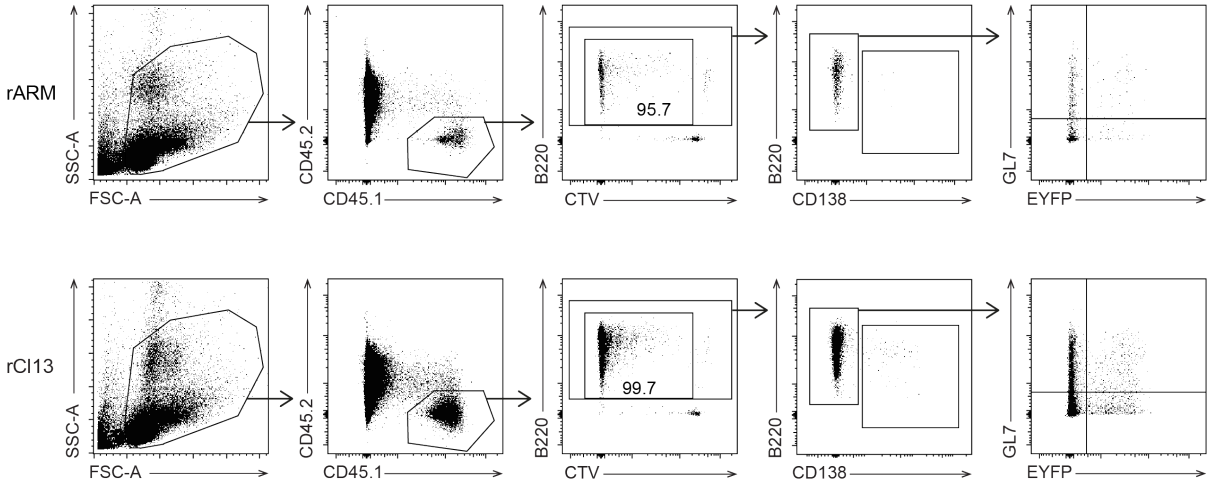

Supplementary Figure 4 (related to Figure 4): Gating strategy for the FACS analyses shown in Figure 4
We infected wt recipient mice with rARM* or rCl13*. On day 20 after infection these animals were given KL25HL-AID^{rep} B cells by adoptive transfer and were administered TAM on day 20 and 23 as shown in Fig 4A. The gating strategy as displayed was used to determine EYFP and GL7 expression by the transferred KL25HL-AID^{rep} B cells. Numbers in FACS plots indicate the percentage of CTV^{lo} cells amongst transferred B220^{+} cells. Representative FACS plots are shown.

Supplementary figure 5

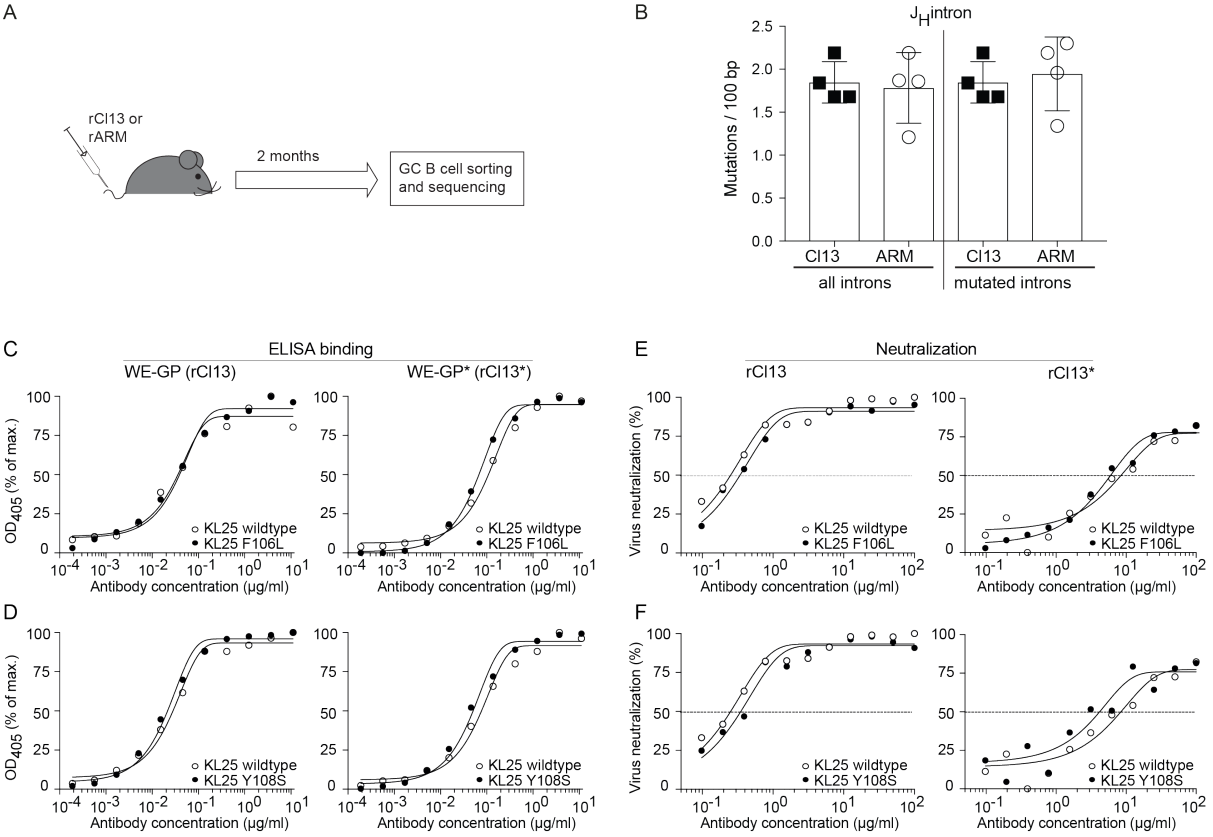

Supplementary Figure 5 (related to Figure 5): Comparable mutational load in V_H introns of GC B cells from rARM- and rCl13-infected mice and characterization of KL25 variants selected in rCl13* infection

A,B: We infected wt mice with rCl13 or rARM. Two month later we sorted $CD3^-$ IgD^- IgM^- $GL7^+$ $B220^+$ B cells from the spleen for IgH intron sequencing as described in Methods. Schematic of the experimental design (A). Mutations frequencies in the J_H intron are shown as nucleotide changes per 100 bp as calculated for all sequences (left) or for mutated sequences only (right), (B). Bars represent mean +/- SD. Symbols represent individual mice. n = 4, N = 1.

C,D: Binding of KL25 wildtype, KL25-Y108S and KL25-F106L to WE-GP and WE-GP*.

E,F: Neutralization of rCl13 and rCl13* by KL25 wildtype, KL25-Y108S and KL25-F106L. Symbols in (C-F) show the mean of 2 technical replicates. N = 2 (D,E).
